# Towards a “Testis in a Dish”: Generation of Mouse Testicular Organoids that Recapitulate Testis Structure and Expression Profiles

**DOI:** 10.7150/ijbs.89480

**Published:** 2024-01-12

**Authors:** Aviya Stopel, Cheli Lev, Stav Dahari, Or Adibi, Leah Armon, Nitzan Gonen

**Affiliations:** The Mina and Everard Goodman Faculty of Life Sciences and the Institute of Nanotechnology and Advanced Materials, Bar-Ilan University, Ramat Gan, 5290002, Israel.

**Keywords:** Testis, organoids, infertility, sex determination, gametogenesis

## Abstract

The testis is responsible for sperm production and androgen synthesis. Abnormalities in testis development and function lead to disorders of sex development and male infertility. Currently, no *in vitro* system exists for modelling the testis. Here, we generated testis organoids from neonatal mouse primary testicular cells using transwell inserts and show that these organoids generate tubule-like structures and cellular organization resembling that of the *in vivo* testis. Gene expression analysis of organoids demonstrates a profile that recapitulates that observed in *in vivo* testis. Embryonic testicular cells, but not adult testicular cells are also capable of forming organoids. These organoids can be maintained in culture for 8-9 weeks and shows signs of entry into meiosis. We further developed defined media compositions that promote the immature versus mature Sertoli cell and Leydig cell states, enabling organoid maturation *in vitro*. These testis organoids are a promising model system for basic research of testes development and function, with translational applications for elucidation and treatment of developmental sex disorders and infertility.

## Introduction

The testes are part of the male reproductive system and are responsible for production, storage, and maturation of spermatids, as well as androgen production and secretion throughout life 1, 2. In the mouse, testes develop at embryonic day (E) 11.5 from the bipotential gonad upon the expression of the sex determining gene, *Sry,* and its downstream target gene,* Sox9* 3, 4. The testis is composed of two main compartments: the testis cords and the interstitium. The testis cords are comprised of the somatic Sertoli cells that encapsulate the germ cells (gonocytes). Peritubular Myoid Cells (PM) are located outside the testis cord, and together with Sertoli cells, help to establish the testicular basement membrane, mainly composed of extra cellular matrix (ECM) proteins. Within the interstitium lie the steroidogenic Leydig cells that secrete androgens, as well as endothelial cells, immune cells, and progenitor cells 1, 5. At puberty, Sertoli cells transform from an immature-to-mature cell state, and the testis cords become the seminiferous tubules. During this stage, the gonocytes, which were initially located at the center of the testis cords, migrate to the periphery of the tubules, next to the basement membrane, and are now termed spermatogonial stem cells (SSC). SSCs which reside within their niche are the source of spermatogenesis in adulthood throughout life 6. Sertoli cells are the only somatic cells being in direct contact with the germ cells, and their roles in nurturing and providing the signals and support needed for germ cell proliferation, maturation, and spermatogenesis are well established 1-3. Sertoli cells are considered to be the “organising hub” of the testis at both embryonic and adult stages 1-3.

Dysfunction of testis development or function leads to various diseases including Disorders of Sex Development (DSD) and infertility. DSD is defined as discordance between the genetic, gonadal, and anatomical sex of an individual with a prevalence of 1:4000 newborns 7, 8. While many genes and pathways have been shown to be involved in DSD, currently only 50% of DSD cases receive a genetic diagnosis following whole exome sequencing (WES), indicating that further study is needed to better understand mammalian sex determination and DSD pathologies. Infertility is defined by the failure to achieve a pregnancy after 12 months or more of regular unprotected sexual intercourse. It is experienced by 1 in 6 people 9, and male infertility accounts for ~50% of cases. While male infertility is easily identified and classified into sub-groups, very little is known about the genetic and environmental mechanisms leading to male infertility 10.

To date, there is no proper *in vitro* system to model the testis at embryonic or adult stages. Culture of primary testicular cells in 2D settings and serum-containing media leads to loss of their normal characteristics, and gene expression patterns rapidly diverge from normal testis 11. Additionally, there are no reliable testicular cell lines that recapitulate the *in vivo* testicular cells 12. This limits studying testis development to *in vivo* models in mice, where the process can be long, expensive, and laborious. Furthermore, while mouse and human sex determination and testicular function systems are generally similar, they are not fully conserved, and many DSD and infertility cases cannot be successfully modelled in mice 13, 14. Hence, the generation of testicular organoids from both mouse and human, primary and stem cell-based approaches, can serve as a useful and powerful platform to explore normal testis development and associated pathologies.

Organoids are three dimensional (3D) structures, cultured *in vitro*, that closely resemble the structure and function of true organs 15. Organoids have been developed to model multiple types of organs such as the intestine, brain, kidney, retina, and others 15. In recent years, several studies described the generation of testicular organoids, mostly from primary testicular cells of human, mouse, pig, and rat (reviewed in 16, 17). These studies used different approaches for culturing the primary testicular cells including decellularized testis, Matrigel, soft agar, as well as microfluidic devices 18-23. While some models were able to recapitulate testis structure and formation of tubule like-structure, preservation of germ cells, and even mild entry to meiosis, most failed to show preservation of the organoids for prolonged periods and did not assess gene expression profiles in comparison to *in vivo* testis.

Here, we employed two transgenic mouse strains that allow us to track the presence and state of Sertoli cells, and explored various culture and media conditions to generate testicular organoids from primary testicular cells. We demonstrate that transwell inserts, which provide a gas-liquid interphase, are optimal for generating testicular organoids. These organoids, formed from primary testicular cells of neonatal pups, can be cultured *in vitro* for 9 weeks while maintaining structure and gene expression profiles that closely resemble the *in vivo* testis at corresponding stages. These organoids preserve the presence of all major somatic cell types and germ cells, and organise into tubule-like structures and interstitial area. We further show that these settings can also be used for culturing embryonic testis-derived organoids with tubular structures. We developed defined media compositions that can promote the immature or mature cell states of Sertoli cells and Leydig cells, and hence may provide a platform for *in vitro* maturation of organoids. Lastly, we show first indications that these organoids can support entry of SSC to meiosis.

## Results

### Culturing primary testicular cells in 2D using serum-based or defined media

We aimed to explore the optimal culture and media conditions for the generation of testicular organoids. As Sertoli cells are known to be crucial somatic cells in the testis that orchestrate gonadal specification and organisation 1, screenings were performed using two reporter mouse lines that harbour a fluorescent protein that specifically labels Sertoli cells. This enables tracking of the state of Sertoli cells, and expression levels of *Sox9*, one of the key markers of Sertoli cells, within the organoids over time. The first mouse line is the TESCO-CFP strain that contains the *Sox9* testis-specific enhancer, TESCO, upstream of the CFP reporter 24. The second mouse line is the *Sox9*-IRES-GFP strain that contains an IRES-GFP downstream of the *Sox9* coding sequence 25. Both reporter mouse lines express CFP or GFP within Sertoli cells of embryonic and adult gonads (Supplementary Figure 1).

**Figure 1:**
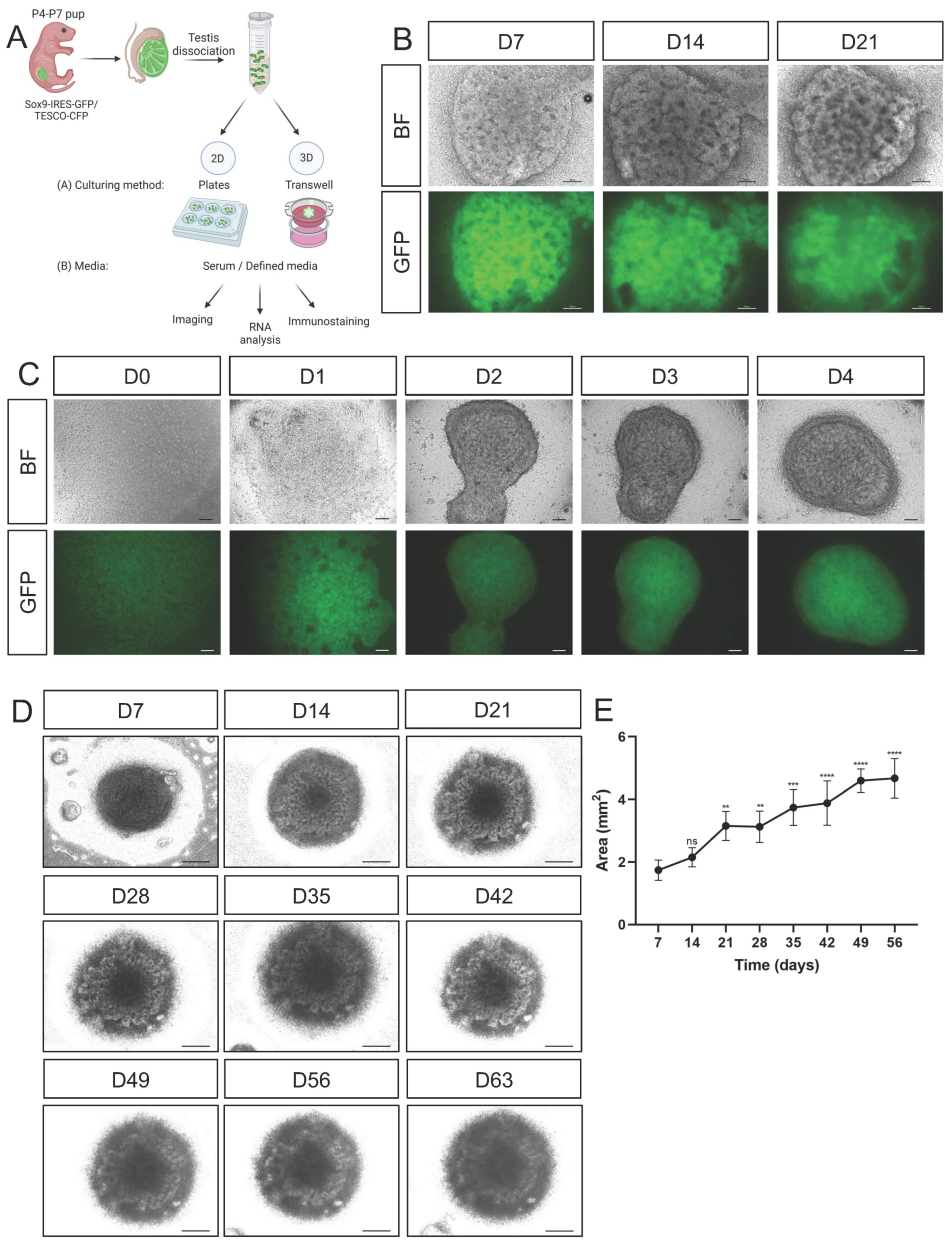
** Establishment of primary neonatal testicular organoids on transwell inserts.** (A) Schematic representation of the experimental design. Primary testicular cells from P4-P7 *Sox9*-IRES-GFP or TESCO-CFP pups were dissociated into single cells and re-assembled under various culturing methods and media compositions. Scheme created using BioRender. (B) Representative bright-field (BF) and fluorescent images of testicular organoids, from *Sox9*-IRES-GFP mice, cultured for 7, 14 and 21 days on transwell inserts. Scale bars, 100 μm. (C) Representative BF and fluorescent images of the organoids forming over the first 4 days of culture. Scale bars, 100 μm. (D) Representative BF images of organoids cultured for prolonged times. Scale bars, 500 μm. (E) Organoids area (mm^2^) over time in culture. Data are presented as mean area ±SEM. All areas are compared to the area of D7 organoids. *P < 0.05, **P < 0.01, ***P < 0.001, and ****P < 0.0001, ns - not significant. *N*=3-4.

For developing testicular organoids, calibrations were performed with postnatal day (P)4-7 neonatal testis rather than embryonic or adult testes. Adult testes were previously shown to lack the ability to form organoids 21, and the smaller relative size of embryonic gonads compared with neonatal gonads translates to fewer testicular cells than what is ideal for large screens. Entire testes from P4-P7 pups of the *Sox*9-IRES-GFP or TESCO-CFP mouse strains were harvested, dissociated into single cells, and plated on either 2D or 3D culture settings in various media compositions. The outcome of the various conditions was assessed by bright field (BF) and fluorescence examination of the cells or organoids, gene expression analysis, and immunostaining (Figure 1A).

Previously described conditions for culturing primary testicular cells in standard 2D culture plates with media containing 2% serum 11 were revisited and used for baseline comparison. As previously demonstrated 11, plating primary testicular cells on plastic gelatin-coated dishes with Ad-DMEM/F12 media containing 2% fetal bovine serum (FBS) is not optimal for maintaining testicular cells (Supplementary Figure 2). The cells adopted a fibroblast-like morphology, had a very dim expression of GFP or CFP and did not form aggregates, characteristic of Sertoli cells 5 (Supplementary Figure 2A). Analysing expression levels of several Sertoli cell markers at days (D)1, 4 and 7 of culture, compared to P4-P7 primary cells that were not cultured *in vitro,* indicated a drastic decrease in genes such as *Amh*, *Cyp26b1*, *Ptgds* and more. Expression of other genes was unstable during the culture period, and differed from the normal levels of expression in neonatal testis (Supplementary Figure 2B).

Most organoid systems developed do not use serum-based media, but rather a tissue-specific defined media, that is devoid of serum, containing instead specific growth factors that are normally present within that tissue 15. We hence aimed to develop a “testis-defined media” (termed from now on “defined media”) containing factors that are normally present in the testis according to literature-based searches (for detailed media composition see Supplementary Table 1). This media contains recombinant human follicle-stimulating hormone (rhFSH) 26, fibroblast growth factor 9 (FGF9) 27, prostaglandin D2 (PGD2) 28, 29, testosterone 30 and activin A 27, 31, all known to be expressed and have key roles in the testis. Interestingly, culturing primary testicular cells on gelatin-coated dishes in defined media resulted in completely different cell morphology compared to the serum containing media used above (Supplementary Figure 3A). The cells formed rounded aggregates expressing CFP/GFP, and thus could be identified as Sertoli cells. Beneath the aggregates there were cells that attached to the plates, forming a “feeder-like layer” to which the aggregates attached (Supplementary Figure 3A). Despite the major morphological change in the cells, gene expression profiles of many Sertoli markers, e.g. *Amh*, *Cyp26b1*, *Dhh* and *Shbg,* were still markedly decreased compared to those of primary cells*.* Other genes, such as *Sox9,* were more stable (Supplementary Figure 3B). This suggest that media change alone, although influential, is not enough to enable proper prolonged culture of primary testicular cells.

Many organoid systems use Matrigel as a coating or scaffolding system for supporting 3D culturing of cells 15. We hence attempted at seeding primary testicular cells on top of various dilutions of Matrigel and compared it to cells grown in wells coated with gelatin (Supplementary Figure 4A). Bright field and fluorescent microscopy demonstrated that the cells were unable to form aggregates and presented very dim CFP expression. qPCR analysis demonstrated a low expression of many Sertoli markers (Supplementary Figure 4). Thus, seeding on top of Matrigel could not support testicular cell growth in culture.

### Culturing primary testicular cells on transwell inserts with defined media

As culturing in various 2D culture conditions did not seem optimal in terms of cell morphology, we sought a 3D culture system that would better support testicular organoid formation and maintenance. Several organoid systems, including kidney, lung, liver, and cerebral organoids, use transwell inserts 32-37. Since the kidney and gonads share common developmental origin from the intermediate mesoderm 38, and they both possess tubular structures, we explored the use of transwell inserts for generating testicular organoids (Figure 1A). To this end, we seeded 3.5x10^5^ primary testicular cells per organoid on transwell inserts and added defined media (See Supplementary Table 1) to the bottom of the well. When cultured on transwell inserts, the cells encounter a liquid-gas interphase with media from the bottom and air from the top. Quite remarkably, beautiful testis organoids were formed that presented clear tubular structures and very strong CFP or GFP expression that persisted for 21 days in culture (Figure 1B and Supplementary Figure 5).

To better assess the early stages of organoid formation, organoids were seeded and examined immediately after seeding and for 4 consecutive days. Already one day after seeding, organoids started to compact, and by day 2 they formed clearly compacted organoids. Further compaction continued on days 3 and 4 (Figure 1C). These organoids could be cultured for up to 9 weeks (D63) while still maintaining compacted organoid morphology with clear tubular structures (Figure 1D). After 9 weeks in culture, however, they started to collapse. Measurement of the area of the organoids indicated that they continued to grow in size during the entire 9-week culture period (Figure 1E).

### Characterising gene expression of testicular organoids cultured on transwell inserts in defined media

**Figure 2:**
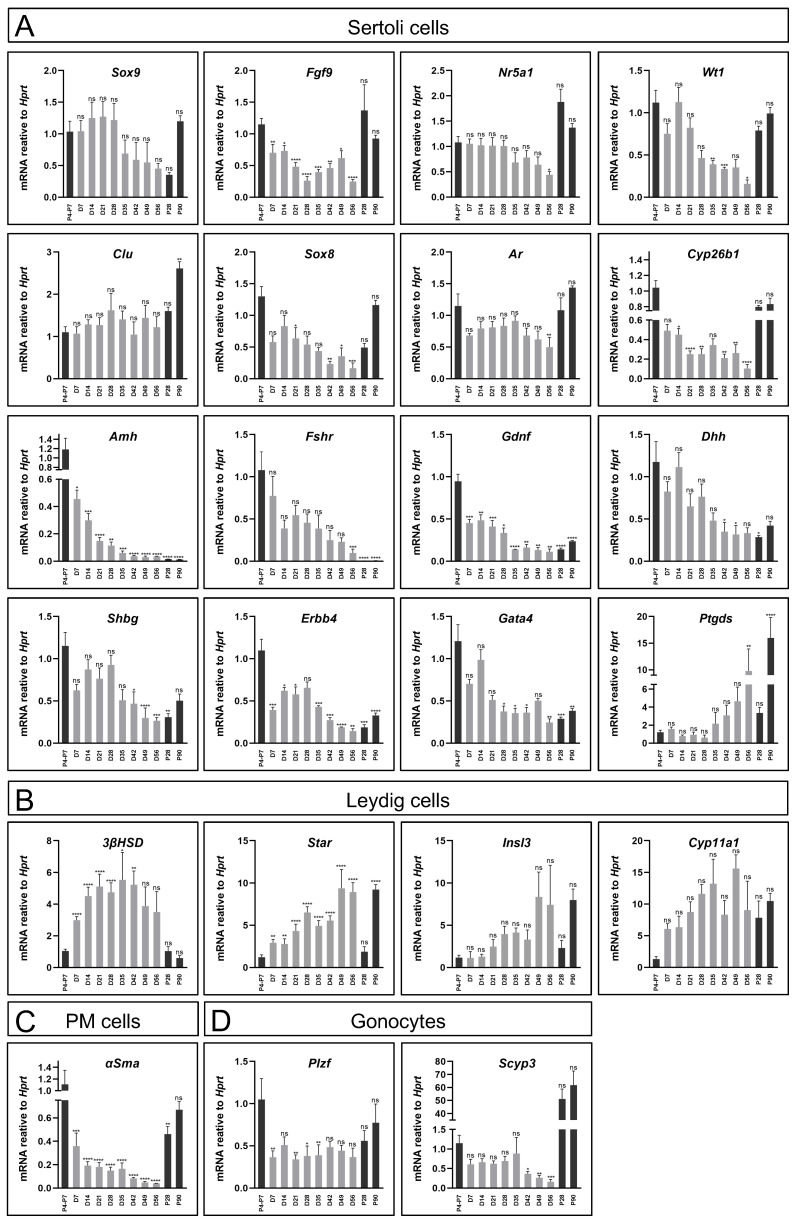
** Comparison of gene expression profile of cultured organoids, neonatal and adult testes.** (A-D) Quantitative RT-PCR was performed on mRNA extracted from neonatal or adult testes, or TESCO-CFP organoids cultured for the indicated number of days assessing Sertoli cell markers (A), Leydig cell markers (B) or other testicular cells markers (C-D). Gene names are presented in the title. D denotes culture day of organoids, grey. P denotes days postpartum (dpp) of *in vivo* testis, black. Data are presented as mean 2^-ΔΔCt^ values ±SEM normalized to the housekeeping gene *Hprt*. *P < 0.05, **P < 0.01, ***P < 0.001, and ****P < 0.0001, ns- not significant. *N*=3-12.

Gene expression profiles of the organoids were assessed and compared to *in vivo* testis, first focusing on Sertoli-specific markers, as Sertoli cells are the organising hub of the testis. Gene expression was measured at various stages of the 8-week organoid culture period, compared to P4-P7 testis, as the reference point from which the organoids were generated, P28 testis, which correspond to the endpoint, D21 organoids, and the adult testis at P90. As evident in Figure 2A, many of the Sertoli-specific markers, i.e. *Sox9*, *Nr5a1*, *Wt1*, *Clu* and *Ar* displayed similar levels of gene expression in the organoid culture as that of the *in vivo* testis throughout the 8-week culture. Interestingly, some Sertoli markers varied in expression in neonatal testis versus adult testis; *Amh, Fshr, Gdnf, Gata4,* and others, decreased in expression as the testis matured, while other genes, like *Ptgds,* showed the opposite pattern. Quite remarkably, the culture of organoids over the 8-week period mimicked these expression trends, presenting decrease gene expression in *Amh, Fshr, Gdnf, Gata4* and increased expression of *Ptgds* over time (Figure 2A).

Since the organoid system was composed of an entire testicular cell mixture, and not only Sertoli cells, expression of several markers of other gonadal cell types were also analysed (Figure 2B-D). As for Leydig cell markers, while *3ßHsd* expression was significantly higher than that of *in vivo* testis, other Leydig markers as *Star*, *Insl3* and *Cyp11a1* exhibited levels that are more comparable to the *in vivo* testis. This indicated that Leydig cells were present and well maintained throughout the 8-week culture period (Figure 2B). *α-Sma,* a marker of PM cells, was also expressed, albeit at lower levels than *in vivo* testes (Figure 2C). Finally, analysis of two gonocytes markers, *Plzf* and *Scyp3*, which are expressed in meiotic cells*,* indicated that they are also expressed in the organoid system (Figure 2D).

Altogether, this gene expression analysis suggests that organoid culture on transwell devices with defined media can support prolonged culture and survival of multiple testicular cell types and maintain gene expression profiles that resemble the *in vivo* testis.

### Testicular organoids cultured on transwell inserts organise into tubular structures reminiscent of the testis

**Figure 3:**
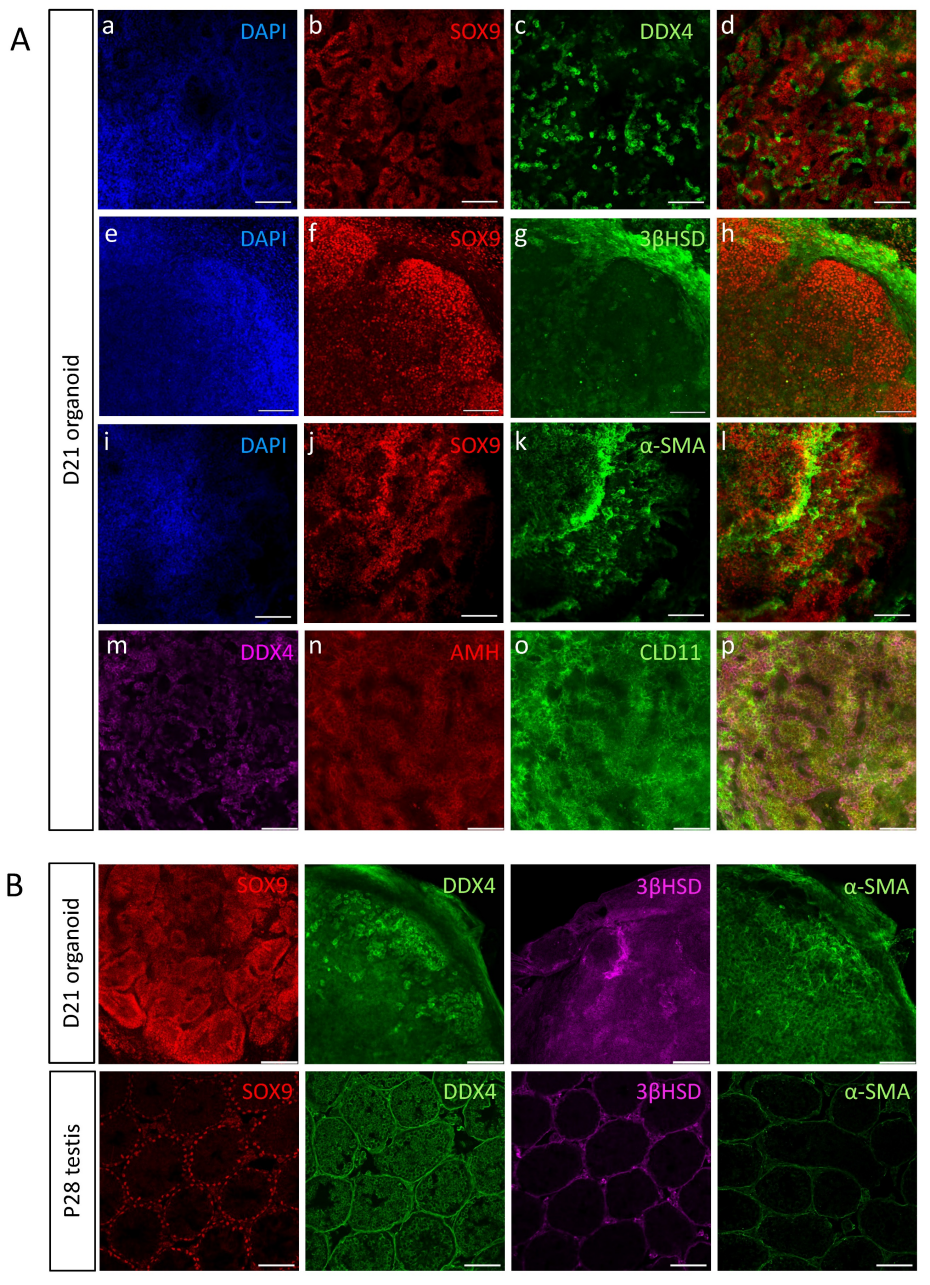
** Testicular organoids present tubular structures and preserve main gonadal cell types.** (A) Whole mount co-immunostaining of markers for the major types of testicular cells in organoids cultured for 21 days. SOX9, AMH, CLD11 (Sertoli cells), DDX4 (Gonocytes), 3ßHSD (Leydig cells), α-SMA (PM cells). Images at right are merged views of all channels. Scale bars, 100 μm. (B) Whole mount immunostaining of markers for the major types of testicular cells in organoids cultured for 21 days compared to sections of P28 testis. Scale bars, 100 μm.

The testes are composed of two main compartments, namely the testis cords that later become the seminiferous tubules and contain Sertoli cells and gonocytes, and the interstitial area, composed mostly of Leydig and PM cells 5, 39. To explore whether all major testicular cell types are present in the organoids and determine their spatial organisation, day 21 (D21) organoids were immunostained with various markers of the different cell types (Figure 3A). Immunostaining of whole-mount D21 organoids with SOX9, AMH, CLD11 (Sertoli cells), 3ßHSD (Leydig cells), DDX4 (gonocytes) and α-SMA (PM cells) indicated that all major testicular cell types are present within the organoids for a period of 21 days in culture (Figure 3A). As the organoids are spherical in shape, multiple z-stack images of whole-mount-stained organoids were acquired, generating movies of these organoids with the various co-stainings (Supplementary movies 1-6). Remarkably, the organoids presented with spatial organisation that closely resembled the *in vivo* testis. Sertoli cells formed numerous tubular structures and the DDX4 positive cells were located next to Sertoli cells and within the tubules. Apparently, the gonocytes had a tendency of localizing towards the outer side of the tubules, but adjacent to Sertoli cells (Figure 3A a-d, Supplementary Movie 1). 3ßHSD-positive Leydig cells were located outside of the SOX9-positive tubules, in an area that corresponds to the interstitial area (Figure 3A e-h, Supplementary Movie 2). α-SMA-positive PM cells were located close to Sertoli cells, yet it was less clear if they are indeed located outside of tubules as they should normally be (Figure 3A i-l, Supplementary Movie 3). Expression of AMH and CLD11 was strong and indicated the presence of tubular structures composed of Sertoli cells, and containing DDX4-positive germ cells (Figure 3A m-p, Supplementary Movie 4).

To better analyse the spatial organisation of testicular organoids and compare it to that of *in vivo* testis, whole-mount immunostaining of D21 organoids was performed and compared to immunostaining of P28 testis (D21 organoid corresponds to P28 as it was harvested at P7 and cultured *in vitro* for 21 days) (Figure 3B). Staining with SOX9 Sertoli cell marker clearly showed the formation of tubules, similar to these of the testis. Analysis of movies composed of multiple z-stacks of whole-mount organoids confirmed the formation of tubules by both SOX9 (Supplementary Movie 5) and DAPI staining (Supplementary Movie 6). DDX4, which marks gonocytes, was strongly expressed and gonocytes seemed to aggregate together. 3ßHSD immunostaining indicated localization outside of tubules, similar to *in vivo* testis. α-SMA on the other hand, despite being strongly expressed in the organoids, did not exhibit specific localization outside of tubules, as normally would be expected (Figure 3B).

Altogether, this data indicates that the *in vitro* testicular organoids form compartments and structures that closely resemble the structure of the *in vivo* testis, and preserve all major cell types for prolonged periods of time.

### Generation of testicular organoids from embryonic testis

**Figure 4:**
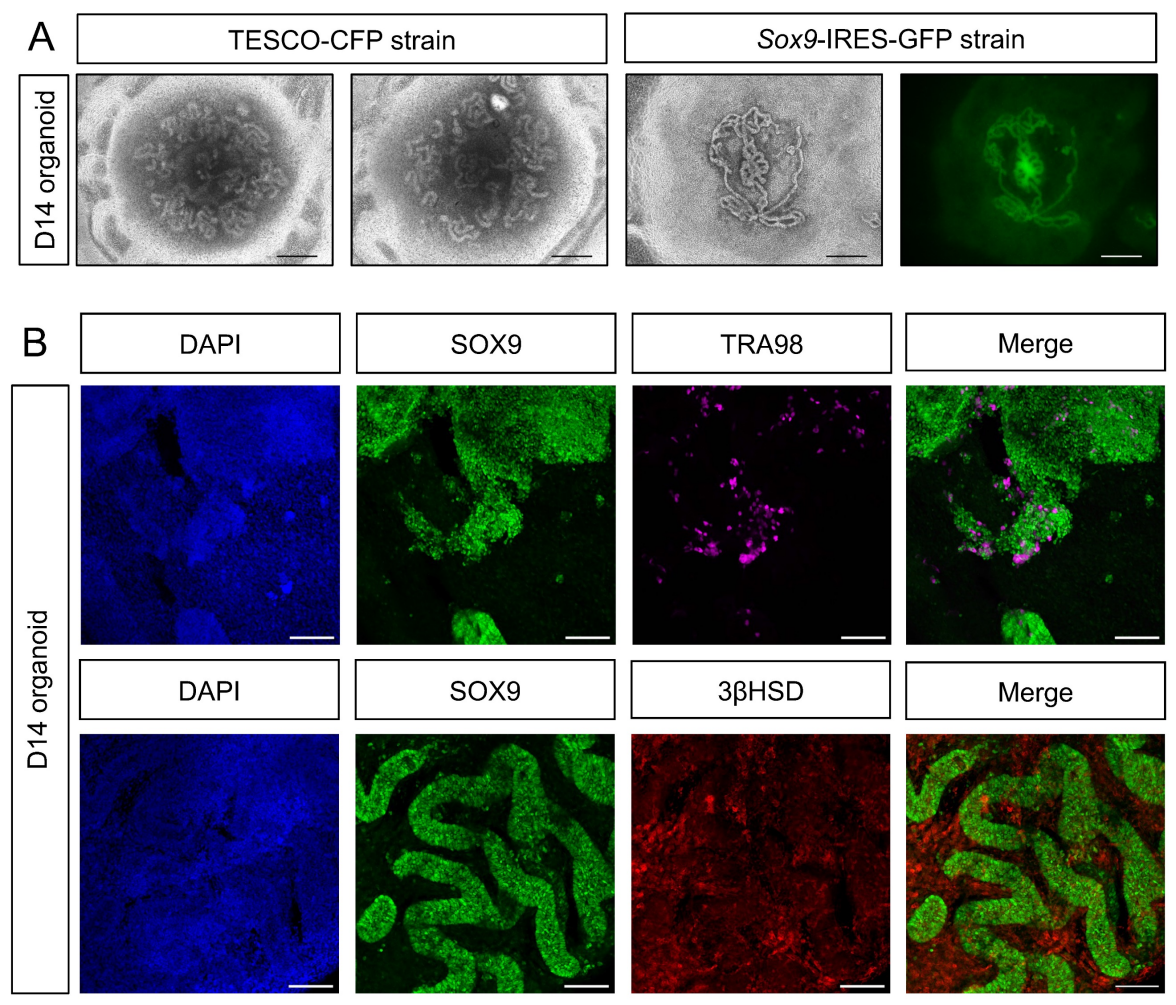
** Establishment of organoid culture from embryonic testes.** (A) Representative BF and fluorescent images of organoids harvested from E12.5-E14.5 embryonic testes and cultured for 14 days. The two left pictures (TESCO-CFP) were cultured on defined media and the two right ones (*Sox9*-IRES-GFP) on immature media. Scale bars, 500 μm. (B) Whole mount immunostaining of markers for the major types of testicular cells in embryonic organoids cultured for 14 days. SOX9 (Sertoli cells), TRA98 (Gonocytes), 3ßHSD (Leydig cells). Scale bars, 100 μm.

Despite the relative convenience of generating organoids from neonates, as opposed to embryos, which require harvesting from timed pregnant females, yielding smaller testis with fewer cells, the former are limited in that many of the disorders related to testis development and dysfunction occur at the embryonic stages (DSD). Thus, for studying DSD, organoids derived from embryos are preferred. Hence, we applied the same settings established above for neonatal testis organoids for generation of testis organoids from embryonic gonads. To this aim, testes were harvested from E12.5-E14.5 embryos of either TESCO-CFP or *Sox9*-IRES-GFP mouse strains, dissociated into single cells and seeded on transwell inserts in defined media. Embryonic-derived testicular cells formed organoids with very pronounced and clear tubular structures, much clearer than these observed using neonatal testis. Figure 4A presents three different embryonic testis organoids after 14 days in culture. The right-hand panel displays the GFP fluorescence from organoid derived from the *Sox9*-IRES-GFP strain. GFP was present within the tubules, indicating that the tubules are formed from Sertoli cells (Figure 4A).

To better characterize the embryonic testicular organoids that were cultured *in vitro* for 14 days, whole mount D14 organoids were immune-stained for markers of various gonadal cell types (Figure 4B). Organoids strongly stained for SOX9; these SOX9-positive cells formed the tubules. TRA98, which marks gonocytes, was also expressed, indicating the presence of germ cells. Furthermore, 3ßHSD was expressed and predominantly located outside of the SOX9-positive tubules, within the interstitium, as expected of Leydig cells (Figure 4B). This data suggests that the transwell system, coupled with defined media, is suitable for the formation of both embryonic and neonatal testicular organoids that preserve most gonadal cell types for prolonged periods and highly resemble testicular compartments and structures.

### Developing media compositions to support the immature and mature states of Sertoli cells

Having shown that the transwell system can support formation of embryonic and neonatal testis organoids, we sought to also culture mature testis in these settings, which would enable studying disorders of the adult testis, such as infertility. Testicular cells were harvested from P90 mature testis and seeded on transwell inserts. In contrast to the embryonic and neonatal testis, adult testicular cells were not capable of forming organoids (Supplementary Figure 6). This is consistent with previous failed attempts at 3D culturing of adult testis organoids 21. As an alternative approach, we attempted to mature neonatal testis organoids to the adult testis state using defined media modification.

**Figure 5:**
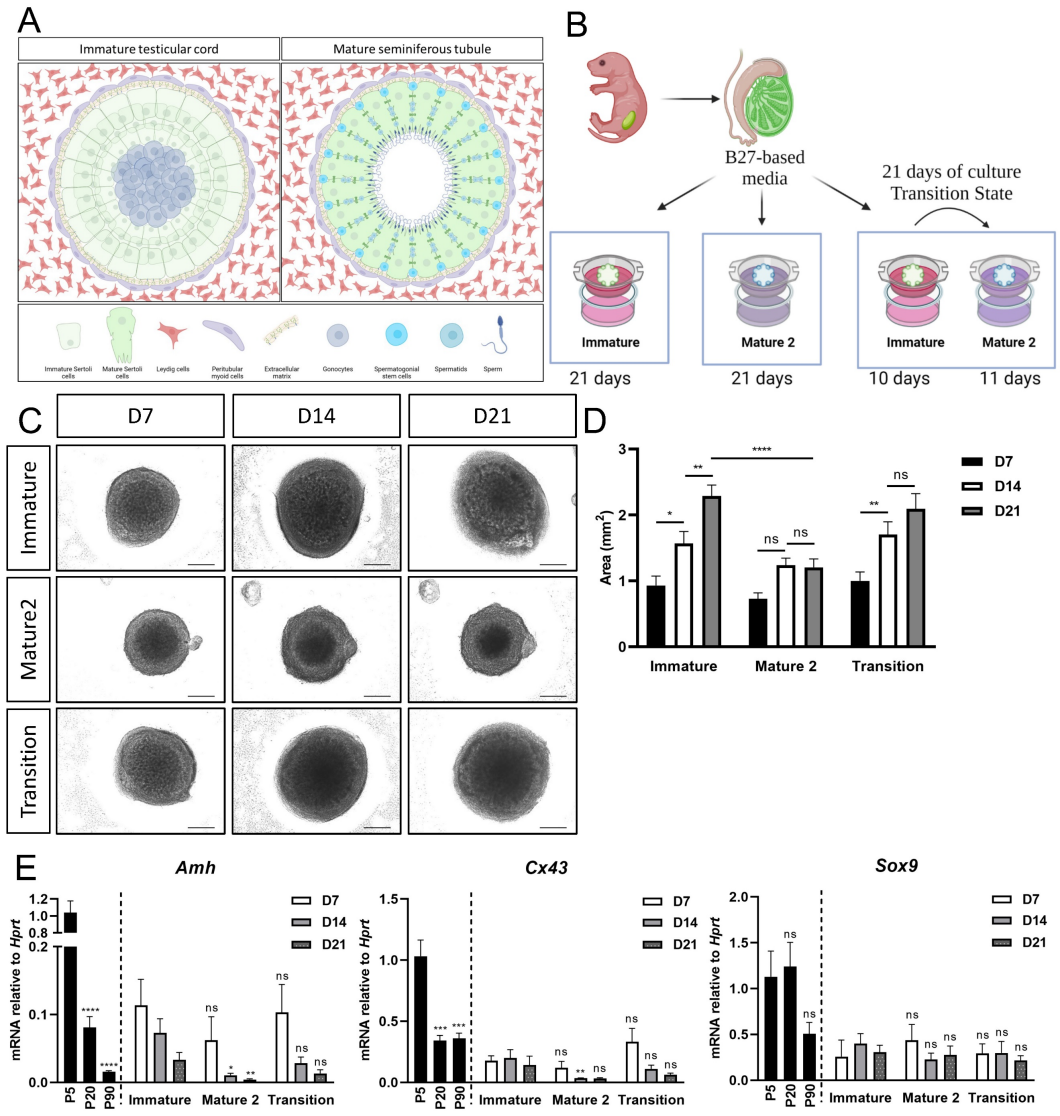
***In vitro* maturation of neonatal organoids in different media compositions.** (A) Schematic representation of pre-pubertal (left side), and adult testis (right side). Testicular cords of the pre-pubertal testis are surrounded by peritubular myoid and Leydig cells in the interstitial compartment. Gonocytes are located at the center of the cords, surrounded by immature Sertoli cells. Post puberty and in adulthood, seminiferous tubules are surrounded by peritubular myoid cells and mature Leydig cells. Germ cells, known as spermatogonial stem cell (SSC) at this point, are situated at the basal side of the tubules, near the basement membrane. As SSC differentiate and undergo meiosis, they migrate from the basal side to the luminal side of the seminiferous tubules. Scheme created using BioRender. (B) Schematic representation of the experimental design. Primary testicular cells from P4-P7 *Sox9*-IRES-GFP or TESCO-CFP mice were dissociated into single cells and re-assembled on transwell inserts and cultured in either immature or mature 2 media for 21 days. A “transition” protocol was also employed wherein organoids were cultured for the first 10 days in immature media and then transitioned to mature 2 media for an additional 11 days, for a total culture period of 21 days. Figures created using BioRender. (C) Representative BF images of organoids cultured with immature or mature 2 media, or transition protocol for 21 days. Scale bars, 500 μm. (D) Organoids area over time of organoids cultured with immature, mature 2 or transition protocol for 21 days. Data are presented as mean area ±SEM. *P < 0.05, ****P < 0.0001, ns - not significant. *N*=9-14. (E) Quantitative PCR analysis of Sertoli cell markers in organoids cultured over time with immature, mature 2 or transition protocol for 21 days, compared to P5, P20 and P90 *in vivo* testis. Data are presented as mean 2^-ΔΔ*C*t^ values ±SEM normalized to the housekeeping gene *Hprt*.*P < 0.05, **P < 0.01, ***P < 0.001, and ****P < 0.0001, ns - not significant. *N*=3-6.

At embryonic stages, the testis is composed of testis cords containing gonocytes, surrounded by immature Sertoli cells and peritubular myoid cells. Immature Sertoli cells have native capacity to proliferate (Figure 5A). At puberty, gonocytes migrate towards the basal lamina and become SSC. Sertoli cells undergo maturation, which promotes their polarization and establishment of the blood-testis barrier. The large polarized Sertoli cells contact spermatids of different stages as the latter undergo meiosis (Figure 5A).

Previous studies have identified many secreted molecules, hormones, and signalling pathways that support either the immature (proliferative) state of Sertoli cells or their mature state that establishes the blood-testis barrier and promotes spermatogenesis. Factors that maintain and enhance the immature state include FSH, Insulin like Growth Factor I (IGF-1), Activin A and FGF9. Factors that promote the mature Sertoli state include Androgens, Thyroid Hormones (TH) and Retinoic Acid (RA) 40. Based on these, we developed three different media compositions: one to support the maintenance of the immature Sertoli state (Immature medium), and two alternative media to promote the maintenance of mature Sertoli cells (Mature 1 and Mature 2 media). The various media compositions were tested on either B27- or Knockout serum replacement (KSR)-based media (Supplementary Figure 7A and Supplementary Table 1). B27 was chosen as it is a basic addition in many types of organoid defined media 41, and KSR because it has been added to several types of testicular cells media 42, 43. All media compositions were compared to the original defined media that was used throughout the study (contains B27, for full description see Supplementary Table 1).

To test the ability of the various media compositions to promote immature versus mature Sertoli state, the expression levels of Sertoli markers that differ between the immature and mature states were measured using quantitative PCR (qPCR). To verify that the tested markers are indeed more highly expressed in one state over the other, their expression was first assessed in P5, P20 and P90 whole testis. *Amh*, *Gata4* and *Cx43* are significantly higher at P5 testis compared to adult testis, i.e., mark the immature Sertoli state, while *Gata1* is more highly expressed in adult testis and marks mature Sertoli cells (Supplementary Figure 7B-C). *Sox9* is not significantly altered between the two states and served as a control. Analysis of the expression levels of *Amh*, *Gata4* and *Cx43* in organoids cultured for 14 days in defined, immature, or two types of mature media, all containing B27, indicated that the immature media allowed for higher expression of all three immature state markers, while the two mature media led to significantly lower expression levels. In contrast, analysis of *Gata1* expression resulted in higher expression in both mature media compared to the immature and defined media, although this was not statistically significant. This indicates that the immature media containing B27 is able to promote the immature Sertoli cell state, while the mature media containing B27 are better at promoting the mature Sertoli cell state (Supplementary Figure 7B).

Similar analysis was conducted for organoids grown for 14 days in defined media (with B27), KSR only (no growth factors added), defined KSR, immature and mature media with KSR. Unlike the changes observed in expression of the various markers between the immature and mature media based on B27, no significant changes in expression of *Amh, Gata4, Cx43* and *Gata1* were observed when different combinations of KSR-based media were used (Supplementary Figure 7C). This suggest that the addition of KSR to the media masks the effect of the growth factors added to the media to promote the different cell states.

Next, the size of organoids grown for 14 days with the various B27-based and the KSR-based media compositions was determined. In line with what was seen by qPCR, the immature B27-based media was able to promote the immature state, resulting in significantly larger organoids at D7 and D14, while the two mature B27-based media led to smaller size organoids at D7 and D14 (Supplementary Figure 7D, left side). In contrast, no major change in organoid size was observed when organoids were cultured on KSR-based media on either D7 or D14 (Supplementary Figure 7D, right side). Based on these results, we decided to continue working with the B27-based media.

*In vivo*, the testis remains in their immature state until ~P15, after which there is a transition to the mature Sertoli state and spermatogenesis commences. We thus developed a protocol in which P5 neonatal testes were harvested, cultured in immature media for 10 days (to mimic the P15 stage), and then transferred to the mature media for an additional 11 days, for a total of 21 days in culture (Figure 5B, the “transition state”). As a control, organoids were cultured for 21 days in either immature media or mature media alone.

As a first read-out, the size of organoids was measured at D7, D14 and D21 of culture. Organoids grown only in immature media exhibited a significant growth in size from D7 to D14 and from D14 to D21 (Figure 5C,D). In contrast, organoids cultured in mature 2 media did not exhibit any growth in size between D7-D21. Interestingly, organoids in the transition media exhibited a significant growth in size from D7 to D14, when they were cultured mainly with immature media, and then showed no significant growth in size from D14 to D21, when they were grown in mature 2 media (Figure 5C-D).

Next, *Amh, Cx43* and *Sox9* expression levels were analysed from organoids cultured in the various media for a period of 7, 14 and 21 days. There was no significant change in gene expression for all genes examined at D7 (Figure 5E). At D14, however, expression levels of *Amh* and *Cx43* were significantly lower in organoids grown on mature 2 media, but not in organoids grown on transition media. On day 21, expression levels of *Amh* and *Cx43* appeared lower in organoids cultured in the mature 2 and transition media compared to the immature media, but this was not statistically significant. No change was evident in the expression of *Sox9*, as expected, as this gene does not change much between the immature and mature Sertoli cell states (Figure 5E). Altogether, these data suggests that the immature and mature states of Sertoli cells can be maintained and promoted in organoids by manipulating the media compositions and growth factors added to the media.

### Analysis of the transition media over prolonged in vitro culture period

As the transition media combination seemed favourable and more closely mimics the *in vivo* state, we performed expression analysis (Supplementary Figures 8-9) as well as immunostaining on organoids cultured for up to 9 weeks (D63) on transition media (Supplementary Figure 10). Expression analysis indicated that most Sertoli markers behave similar to what we would expect from *in vivo* testis (Supplementary Figures 8, Figure 2). Markers of Leydig cells, PM cells, as well as gonocytes, were also well maintained (Supplementary Figures 8, Figure 2). As expected, under the transition media, immature Sertoli markers as *Amh*, *Gata4* and *Cx43* decreased with culture time, while *Gata1*, which marks mature Sertoli cells, increased in culture compared to P4-P7 testis (Supplementary Figures 9A).

Similar to the maturation of Sertoli cells in the testis, Leydig cells also undergo maturation as testis mature 44. While immature testis contains Fetal Leydig cells (FLC), these are replaced in adult testis with Adult Leydig cells (ALC). To examine if the transition media also promote maturation from FLC to ALC, we examined expression of genes that were shown to be FLC-specific or ALC-specific 44. Among the FLC-specific genes are the *Gsg1l* and *Crhr1* while an ALC-specific gene is the *Hsd3b6* gene 44. Remarkably, qPCR analysis indicated that the two FLC markers were markedly decreased in culture already by D14 (in which the organoids are already in mature media), while a major increase is seen with the expression of *Hsd36b*, which marks ALC (Supplementary Figures 9B). These finding suggest that the transition media is able to induce maturation also of Leydig cells and not only Sertoli cells.

Next, since the organoids can be maintained for 9 weeks *in vitro* and all staining so far were done on D21 organoids cultured on defined media, we next performed stainings with major gonadal cell type markers on organoids grown on transition media at D7, D35, D49 and D63 (Supplementary Figures 10A-D). These stainings suggest that all gonadal cell types and testicular structures are well maintained for the 9-week culture period.

### Testicular organoids allow entry of SSC to meiosis

**Figure 6:**
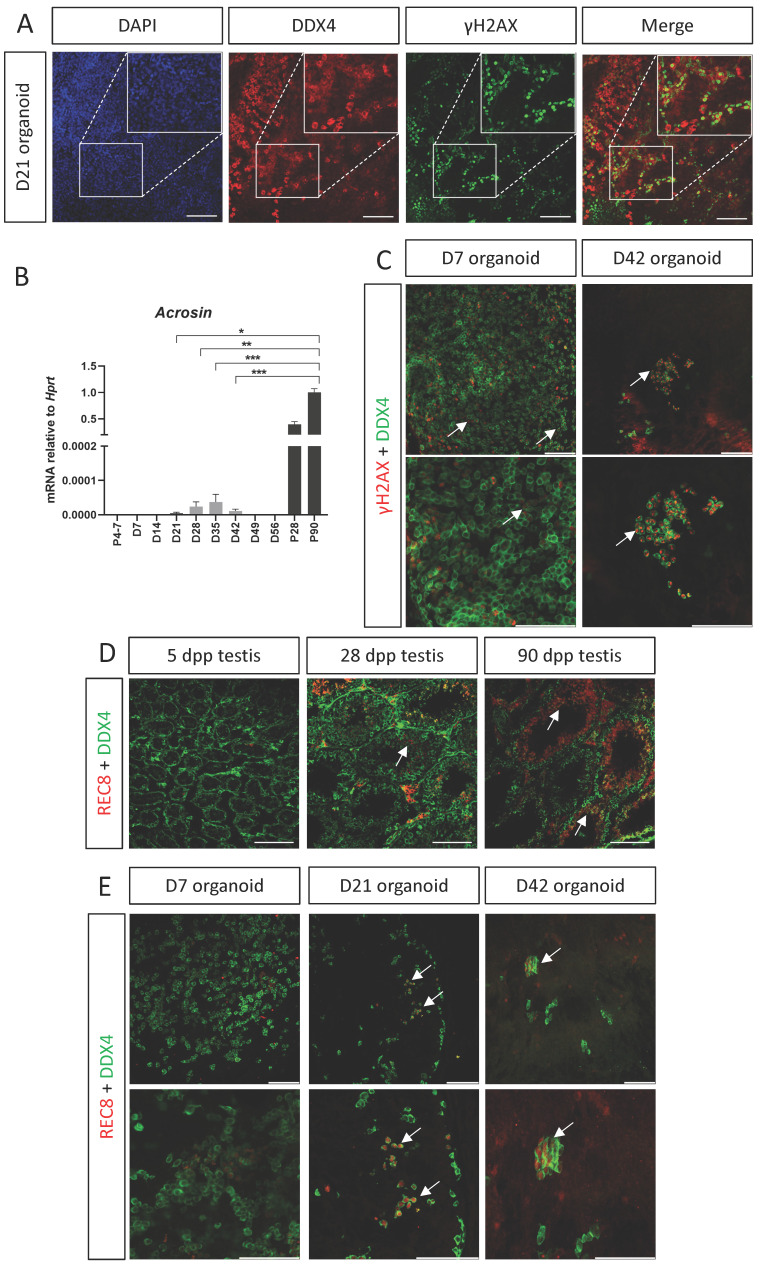
** SSC enter meiosis *in vitro* within testis organoids.** (A) Whole mount immunostaining of γH2AX, a marker for meiotic cells, and DDX4, which marks all germ cells in D21 organoid cultured in defined media. Scale bars, 100 μm. Insets are magnified views of the boxed region. (B) Quantitative PCR analysis of *Acrozin* in testicular organoids over time, cultured in defined media, compared to P5, P28 and P90 *in vivo* testis. Data are presented as mean 2^-ΔΔ*C*t^ values ±SEM normalized to the housekeeping gene *Hprt*. *P < 0.05, **P < 0.01, ***P < 0.001, ns - not significant. *N*=3-6. (C) Whole mount immunostaining of γH2AX and DDX4 on D7 and D42 organoids cultured on transition media. White arrows indicate co-staining of γH2AX and DDX4. Scale bars, 100 μm. (D) Immunostaining of testicular sections of 5, 28, 90 dpp mice. Sections were stained with REC8, which marks a cohesin subunit in meiotic cells and DDX4, which marks all germ cells. REC8 expression is evident only in 28 and 90 dpp in specific seminiferous tubules, indicated by white arrows. (E) Whole mount immunostaining of REC8 and DDX4 on D7, D21 and D42 organoid cultured on transition media. White arrows indicate co-staining of REC8 and DDX4. Scale bars, 100 μm.

One of the main functions of the testis is to support spermatogenesis and haploid sperm production. Spermatogenesis is a long process in which diploid spermatogonial stem cells undergo meiosis to form haploid round spermatids that will undergo spermiogenesis, giving rise to the sperm in its mature form with the acrosome cap on the anterior region of the head. One of the hallmarks of meiosis is formation of double strand breaks (DSB) and homologous recombination of genetic material between sister chromatids 45. To determine whether the organoids support entry of spermatogonial stem cells into meiosis, D21 organoids, first cultured on defined media, were co-stained with the meiotic marker γH2AX, which labels DSB, and DDX4, which marks all stages of SSC and spermatogonia. γH2AX foci are normally present in intermediate and type B spermatogonia and in preleptotene to zygotene spermatocytes. Type A spermatogonia and round spermatids also stain for γH2AX, but as homogeneous nuclear staining 46, 47. Figure 6A demonstrates that some of the DDX4-positive cells are also γH2AX-positive, suggesting that cells may enter meiosis within the organoids* in vitro*. Another indication for the presence of meiosis came from qPCR analysis for the expression of *Acrosin*, a gene specifically expressed at the mature sperm head within the acrosome. While P4-P7 testes do not show any *Acrosin* expression, as meiosis has yet to commence at that stage, P28 and P90 testes demonstrate very high levels of *Acrosin* expression (Figure 6B). Interestingly, *in vitro* testis organoids exhibited low levels or *Acrosin* expression, mostly at D21-D42. Although this expression level is very low compared to *in vivo* testis, its time-dependent presence may indicate the existence of small quantities of fully mature sperm at the later stages of organoid culture (Figure 6B). Next, we analysed whether D7 and D42 organoids, cultured on transition media, also present co-staining of DDX4 and γH2AX. As evident in Figure 6C, while few cells exhibit co-staining at D7 organoids, many are co-stained in D42 organoids.

Although γH2AX marks spermatogonia, it can also mark cells with DSB, hence, to verify that organoid SSC indeed enter meiosis, we analysed the presence of REC8, a subunit of the cohesin complex which function during meiosis 48, 49. We first stained testis sections of 5, 28, and 90 dpp testis with REC8 and DDX4. As expected, no REC8 expression is seen in 5 dpp testis, but substantial REC8 can be seen in 28 dpp and 90 dpp testis section is specific seminiferous tubules, confirming the specificity of the antibody (Figure 6D). Next, we performed whole mount staining with REC8 and DDX4 on D7, D21 and D42 organoids, cultured in transition media. While no overlap is evident at D7 organoids, double positive cells can be seen in D21 and D42 organoids.

Altogether, these finding suggest that *in vitro* organoid culture can support entry of SSC into meiosis, and some may even complete spermatogenesis and form a sperm head containing ACROSIN.

## Discussion

The testis is composed of several cell types that orchestrate complex processes such as sperm production and hormone secretion. These processes require coordination and a high level of regulation and interactions among the different testicular cell types. Despite several attempts, it remains a challenge to model the testis *in vitro* 16, 17. Here, we generated testicular organoids from embryonic and neonatal testis on transwell inserts and showed that they closely resemble the *in vivo* testis in terms of gene expression and spatial organisation. While several other studies that generated testis organoids showed the presence of the various gonadal cell types by immunostaining 18-23, few analysed gene expression, and none compared gene expression to *in vivo* testis. The current study focused mainly on Sertoli cells, since they are considered the ”organising hub” of the testis 50, and for the first time, used fluorescent reporters to track their state. Quite remarkably, the testicular organoid setting maintained proper expression of many Sertoli cell gene markers but also others for the entire 8-week culture duration, to levels comparable with *in vivo* testes at corresponding stages. Moreover, the organoid system was able to capture, and mimic trends of expression changes observed for some genes *in vivo*.

While most of our study was conducted on neonatal testis, we also examined the ability of embryonic and adult testis to form testis organoids. Whereas neonatal testis generated testicular organoids that formed within 2 days, embryonic testes were even better at forming testis organoids and formed tubular structures that were more profound and well-defined than the neonatal testis. Adult testis failed to form any organoids. The inability of adult testis to form testis organoids have been demonstrated before 21. These observations suggest that the more immature Sertoli cells are, the more capable they are at forming testicular organoids and tubular structures. This is consistent with known testis development, in which E11.5-E12.5 Sertoli cells mediate testis cord formation and encapsulation of the gonocytes 1, 39. We hypothesize that once Sertoli cells undergo maturation, they lose the ability to aggregate and mediate tubule formation, explaining the failure of adult testicular cells to form organoids.

Interestingly, our neonatal organoids could be maintained *in vitro* for a period of 9 weeks, in which they grew in size, and after which they collapsed. *In vivo*, the testes contain a substantial blood vessel network that provides constant blood supply at embryonic and adult stages 51, 52. It is possible that without a proper blood supply, the organoids collapse once they reach a certain size. It is hence worth considering actively adding endothelial cells to the culture to promote vascularization of the organoids.

Although the neonatal testicular suspension should contain endothelial cells, normally present in the testis, these may fail to properly organise and form blood vessels. Addition of endothelial cells to other organoid systems such as the brain and kidney has proven to be beneficial 53, 54. Despite the fact that the organoids could be maintained *in vitro* for only 9 weeks, this is still a long enough period of time to enable translational applications. As *in vivo*, the spermatogenesis process last ~35 days in mice 55, a culture period of 9 weeks should theoretically allow enough time for the entire spermatogenesis process to occur *in vitro*.

While several studies aimed to develop testis organoids using various scaffolding approaches, none explored in depth the media composition and its effect on organoid development and function 18-23. It is highly established that the media composition could have profound impact on organoid formation 15. We hence relied on literature search and performed experimental analysis of various media compositions. We namely compared B27-based or KSR-based media as well as examined growth factor and hormone compositions that should favour the immature versus the mature states of Sertoli cells 40. We demonstrate that B27-based media are favourable over the KSR-based media. Indeed, although many testicular cultures employ KSR-based media, others have previously shown a negative effect of KSR on germ cell differentiation 56. Furthermore, we show that our immature media is able to promote expression of genes that are normally specifically expressed at the immature Sertoli and Leydig states while the mature media promotes expression of genes expressed at the mature Sertoli and Leydig states. Likewise, the two media affect organoid size, demonstrating that the immature media can promote proliferation while the mature media cannot. While embryonic and neonatal testis can efficiently form testis organoids, testicular cells at this stage do not normally support spermatogenesis. Considering that adult testis, which normally do support spermatogenesis, are devoid of the ability to form testis organoids, our approach of *in vitro* maturation of neonatal testis is very attractive and may pave the way to allow successful *in vitro* spermatogenesis.

Apart from examining gene expression and structure, it is important to assess organoid functionality. In this study we provide initial analysis of organoid functionality by exploring the expression of *Acrosin*, a mature sperm marker and the presence of γH2AX and REC8 in DDX4-positive spermatids, which suggest entry into meiosis. These analyses provide an indication that spermatids in the organoids can enter meiosis, however additional studies should be performed to fully characterise the scope and efficiency of the *in vitro* spermatogenesis in the organoids. Analysis of the ability of organoids to secrete hormones and other secreted factors should also be assessed. These will be subjects for future studies in the lab.

Organoids can be generated from primary tissues or from pluripotent stem cells-differentiated cells 15, 57. While the testis contains a stem cell population, the SSC, which gives rise to sperm throughout life, it is not known to contain any stem cell progenitors of the somatic lineages 5. Most testicular organoids generated so far, including ours, were produced from primary testicular cells 16-23. We believe that *in vivo* testis represents the optimal tissue to calibrate the ideal conditions needed for the generation of testicular organoids. Recent studies report the ability to differentiate both germ cells and somatic cells of the gonads from embryonic stem cells (ESC) or induced pluripotent stem cells (iPSC). Excellent protocols exist for the generation of primordial germ cell-like cells (PGCLC) 58, 59. Transplantation of these PGCLC into testis lacking endogenous germ cells enabled sperm production that was competent for egg fertilization and eventually, normal pups were born 58. Furthermore, we and others have recently demonstrated the ability to generate early gonadal somatic cells from ESC/ iPSCs 60-62. We envisage that it will be possible to combine PGCLC along with stem cell-derived testicular somatic cells within the organoid system we developed here. It is possible that this system could generate fully “artificial testes” that could be used for research and translational applications. Similarly, Yoshino et al., 62 have recently generated “ovaroids” by the combination of PGCLC along with follicular somatic cells-like cells within an ovary organoid system. They demonstrated that oocytes can be generated in these “ovaroids,” and these can be fertilized by sperm and give rise to healthy, normal pups 62. The generation of stem cell-derived testicular organoids will also enable to model cases of DSD and infertility *in vitro* and will allow the ability to integrate patient-derived variants in one gonadal cell and not another to better understand the mechanisms behind specific patients' variants.

In this study we developed testicular organoids from mice, but it is highly possible that similar settings could be applied to generate testicular organoids from pre-pubertal boys. Development of cancer in pre-pubertal boys followed by chemotherapy and radiation treatments leads to 1 in 3 boys remaining infertile 63. With 85% of young cancer patients surviving to adulthood, this poses a major health concern that needs to be addressed. Currently, no treatments are available to restore male fertility in such patients. These children are often offered testicular tissue storage, and while live births have been reported from preserved ovarian tissues, none have been reported from testicular tissues 63. It will be interesting to explore whether the settings developed here could apply for human testicular tissues, mostly from pre-pubertal boys, as these should contain immature Sertoli cells.

Although organoid research and models have increased substantially over the last decade, the field of testicular organoids is still in its' infancy 16, 17. Yet, there are many possible applications in which testicular organoids can be used for. The first is for research and disease modelling. Malformation or dysfunction of the testis lead to DSD and infertility, two widespread pathologies. Development of testis organoids, and mostly pluripotent stem cell-derived organoids could substantially enhance our understanding on how the testis develop and function. It could allow us to better explore the interaction and cross talk between the different gonadal cells, most notably the germ cells and somatic cells.

Apart from basic research, testis organoids could be a robust and high throughput *in vitro* model to explore the effects of exposure to pharmacological drugs, toxins, endocrine disruptors, viruses, and environmental factors that are known to negatively affect testicular function and male fertility 16, 64. Nowadays, these studies are done at pharmacological companies using *in vivo* animal models. Lastly, if these organoids are able to fully mimic the functionality of adult testis, we would expect them to be able to produce haploid sperm *in vitro*. This ability could be revolutionary and enable infertile patients to have a biological child. Major advances have been developed in recent years with regards to *in vitro* gametogenesis, mostly on the oocyte side 62, 65. We hope that this study, and next to follow, will pave the way to enable us to produce fully functional sperm *in vitro*.

In conclusion, the testis organoids described here represent an advanced *in vitro* model system of the testes. We anticipate that additional improvement and adaptation of the system, including transitioning to human testes, will allow it to serve as a platform to readily explore testicular function and development, and to probe interactions and cross-talk among the different gonadal cells, most notably the germ cells and somatic cells. Furthermore, it can be used to model pathologies related to the testis.

## Materials and Methods

### Mice

All animals were maintained with appropriate husbandry according to Bar Ilan University ethics protocols 57-08-2019 and 61-11-2020. Mouse strains TESCO-CFP 24 and *Sox9*-IRES-GFP 25 were maintained on an F1 (C57BL/6J X CBA) genetic background. Primers used for genotyping are listed in Table S2. Embryos and animals used in this study were either TESCO-CFP homozygotes or *Sox9*-IRES-GFP heterozygotes for GFP.

### Harvesting testicular cells

Testes from P4-7 pups or adult mice were dissected and first dissociated mechanically by cutting into small pieces with forceps. Next, to obtain single cell testicular cell suspension, testicular pieces were incubated with final concentrations of 0.045% Trypsin (Thermo Scientific, 25300062) and 0.25% Collagenase II (Worthington, LS004176) for 30 min at 37°C with gentle shaking every 10 min. Then, 8 ml of DMEM/F12 (Thermo Scientific 12634-010) medium containing 1x penicillin-streptomycin solution (Thermo Scientific, 15140122) and 2mM L-glutamine (Biological Industries, BI-03-020-1A) were added to the suspension and the sample was filtered through a 70 μm cell strainer (Lifegene, CSS010070S). After centrifugation at 300 x g for 5 min at 4°C, cell pellet was resuspended in 10 ml medium, and cell number was counted using Countess II (Invitrogen).

For embryonic organoids, embryos were collected after timed matings at embryonic day E12.5-14.5, with noon of the day of plug designated as E0.5. Testes were harvested, separated from the mesonephros, and enzymatically dissociated as mentioned above for 8 min at 37°C. Then 3 ml of DMEM/F12 medium containing 1x penicillin-streptomycin and 2mM L-glutamine were added, cells were centrifuged at 300 x g and cell pellet was resuspended in 1 ml medium and cell number was counted.

### Culturing testicular cells in 2D

Prior to seeding, 6-well plates (Corning 3516) were coated either with 0.2% gelatin (Sigma-Aldrich G9391) for at least 15 min at room temperature, or with Geltrex (Thermo Scientific A1413201) diluted in cold DMEM/F12 media at three different dilutions (1:100, 1:50, 1:20) for 60 min at 37°C followed by 30 min at room temperature. When using serum-based media, cells from ½ testis of one P4-7 pup were seeded per well of a 6-well plate (roughly 0.6x10^6^ cells). When using defined medium, cells from one testis were seeded per well of a 6-well plate (roughly 1.2x10^6^ cells). Different amounts of cells were seeded in the serum-based or defined media due to the different expansion rate of the cells under both conditions. Cells were cultured for up to 7 days at 34°C in a 5% CO_2_ incubator. Media was replaced every 3-4 days. For media compositions see Table S1.

### Organoids generation and culture

Testicular cells were plated on Transwell 0.4 μm pore polyester membranes (Corning 3450 / Greiner 657641) at 2-2.5x10^5^ cells per organoid for embryonic organoids, and 3.5x10^5^ cells per organoid for neonatal and adult organoids. 7-9 organoids were seeded as pellets on one transwell insert with gaps in between the organoids. Organoids were cultured with the specified media (for media compositions see Table S1) at 34°C in a 5% CO_2_ incubator. Media was changed every 3-4 days from below the transwell inserts. For transition experiments, organoids were cultured for 10 days in immature media (Table S1) and then media was replaced to mature 2 media (Table S1) for an additional 11 days with a duration of 21 days in total.

### RNA isolation, cDNA preparation and qRT-PCR

Total RNA from mouse testis or organoids was extracted using TRIzol™ Reagent (Thermo Scientific, 15596026) according to the manufacturer's protocol. Normally, 3-4 organoids were used for an RNA sample. RNA yield was quantified using a NanoDrop spectrophotometer, and 1500 ng RNA was treated with RQ1 DNase (Promega M610A) and used to synthesize cDNA using SuperScript™ III Reverse Transcriptase (Thermo Scientific 18080085). qRT-PCR reactions were performed in duplicate using Power SYBR Green PCR Master Mix (Thermo Scientific 4367659) and 140 nM each of forward and reverse primers and analyzed on the QuantStudio 1 Real-Time PCR System (Thermo Scientific). Primers used are listed in Table S3.

### Imaging of organoids and measurement of organoids size

All bright field and fluorescent images of organoids were taken using the Nikon Eclipse Ts2R microscope. For organoids size assessment, bright field images were taken on a Nikon Eclipse Ts2R microscope, and the organoid area (*N*= 3-14 organoids) was measured using NIS-Elements D software.

### Statistical analysis

Statistical analyses were carried out using Prism 9 software (GraphPad). The analyses used were either one-way ANOVA followed by Dunnett's, Kruskal-Wallis followed by Dunn's or Brown-Forsythe and Welch followed by Dunnett's post hoc tests, depending on the dataset. When both media compositions and times were compared, two-way ANOVA followed by Tukey's/Dunnett's was used. All experiments were repeated at least three independent times.

### Frozen sections and whole mount immunostaining

Testes were harvested and fixed overnight in 4% PFA (Sigma P6148) at 4°C while rotating. Then, testes were washed three times in PBS + 0.1% Triton X-100 (PBST) (Sigma 9002-93-1) and incubated at 4°C in 20% sucrose (Fisher BioReagents BP220-1) until embedded in OCT (Leica Biosystems 14020108926). Embedded samples were sectioned at 10 µm-thick sagittal sections using a cryostat (Leica Biosystems CM3050S). Antigen retrieval was performed with DAKO (Target retrieval solution, Agilent S1699) at 65°C for 30 min. Samples were then blocked in PBST containing 10% donkey serum (Sigma Aldrich D9663) for 1 h and incubated with primary antibodies (diluted in PBST containing 1% donkey serum) overnight at 4ºC (All primary and secondary antibodies used are listed in Table S4). After three washes with PBST, secondary antibodies and 4',6-diamidino-2-phenylindole (DAPI, Molecular Probes, Thermo Scientific D-1306) were added for 1 h at room temperature. Slides were then washed, dried, and mounted (Polysciences 18606).

For whole mount immunostainings, organoids were fixed in 4% PFA for 20 min at room temperature. Blocking was performed in 5% donkey serum in PBS + 0.3% Triton X-100 solution for 2-3 h at room temperature. Primary antibodies were incubated in PBS + 0.3% Triton X-100 solution supplemented with 10% donkey serum overnight at 4ºC. Following six PBS + 0.3% Triton X-100 washes, organoids were incubated with secondary antibodies and DAPI overnight at 4ºC. They were then washed in PBS + 0.3% Triton X-100 and transferred onto glass slides with a mounting solution. All primary and secondary antibodies used are listed in Table S4. Images were obtained with a Leica Microsystems SP8 confocal microscope.

## Supplementary Material

Supplementary figures and tables, movie legends.

Supplementary movie 1.

Supplementary movie 2.

Supplementary movie 3.

Supplementary movie 4.

Supplementary movie 5.

Supplementary movie 6.
