# Supplementary material for: Towards a “Testis in a Dish”: Generation of Mouse Testicular Organoids that Recapitulate Testis Structure and Expression Profiles
Source: Int J Biol Sci. 2024 Jan 12;20(3):1024–41. doi: 10.7150/ijbs.89480 (PMC10797687; doi:10.7150/ijbs.89480)
Supplement: Supplementary file 1 — Supplementary figures and tables, movie legends. [file ijbsv20p1024s1.pdf]

Supplementary Materials for

Towards a "Testis in a Dish": Generation of Mouse Testicular Organoids that Recapitulate Testis Structure and Expression profiles

Aviya Stopel *et al.*

*Corresponding author. Email: Nitzan.Gonen@biu.ac.il

This PDF file includes:

Figs. S1 to S10
Tables S1 to S4

Other Supplementary Materials for this manuscript include the following:
Movies S1 to S6

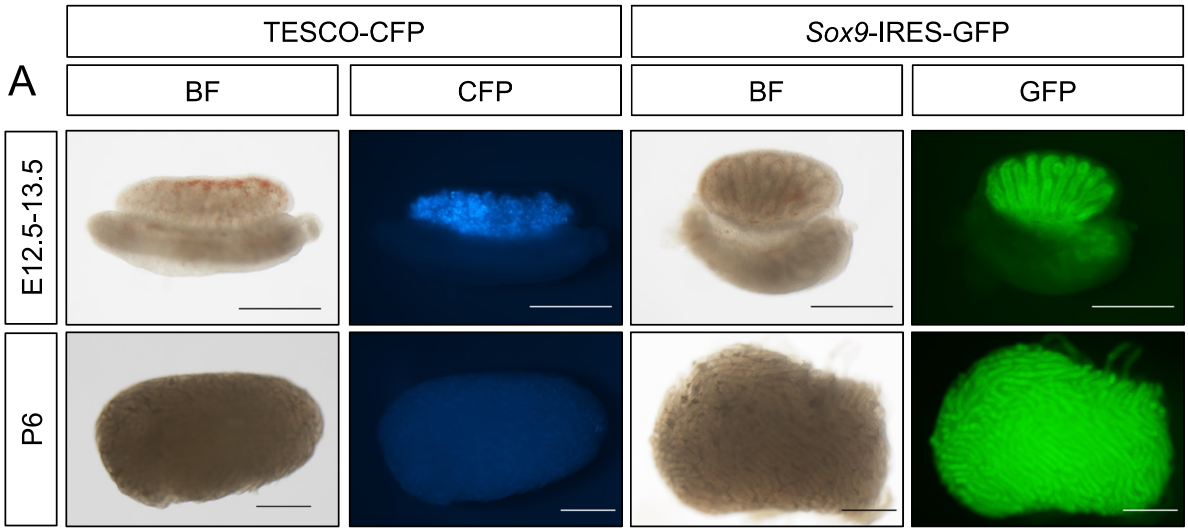

Supplementary Figure 1. Sertoli cell-specific reporter mouse lines used for testicular harvest. Bright-field (BF) and fluorescent images of testes from either E12.5-13.5 embryos or P6 pups harvested from *Sox9*-IRES-GFP or TESCO-CFP mice. Scale bars, 500 μm.

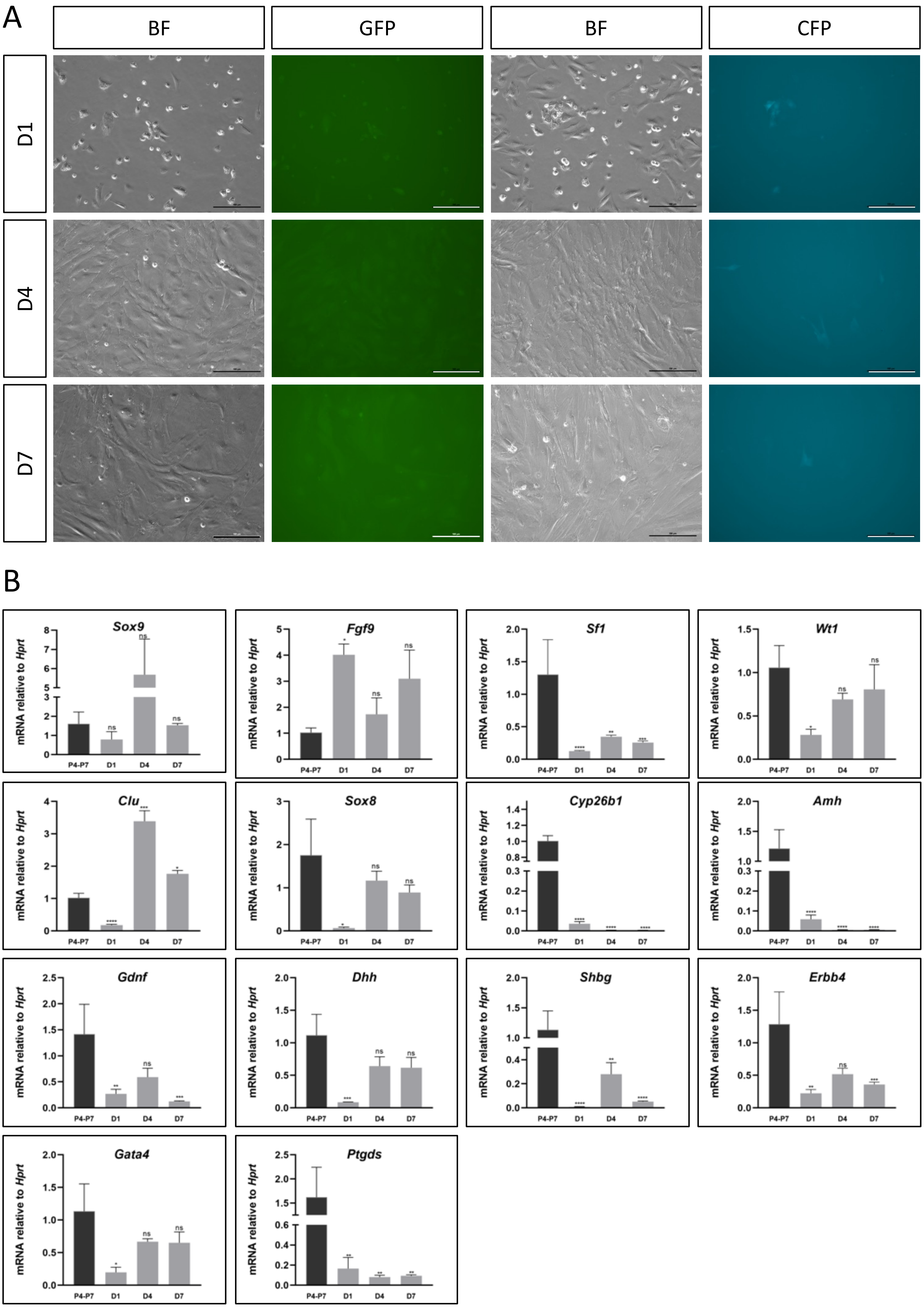
A
BF
GFP
BF
CFP
D1
D4
D7
B
Sox9
Fgf9
Sf1
Wt1
Clu
Sox8
Cyp26b1
Amh
Gdnf
Dhh
Shbg
Erbb4
Gata4
Ptgds
mRNA relative to Hprt
P4-P7
D1
D4
D7
ns

Supplementary Figure 2. Testicular cells cultured in 2D with serum-based media. (A) BF and fluorescent images of testicular cells harvested from either *Sox9*-IRES-GFP or TESCO-CFP pups. Cells were cultured for 7 days on 0.2% gelatin-coated dishes in Ad-DMEM/F12 media supplemented with 2% FBS. Scale bars, 100 μm. (B) Quantitative PCR (qPCR) analysis of testicular cells from TESCO-CFP mice, cultured for 1-7 days in serum-based media. Data are presented as mean $2^{-\Delta\Delta Ct}$ values ±SEM normalized to the housekeeping gene *Hprt*. *$P < 0.05$, **$P < 0.01$, ***$P < 0.001$, and ****$P < 0.0001$, ns - not significant. N=3.

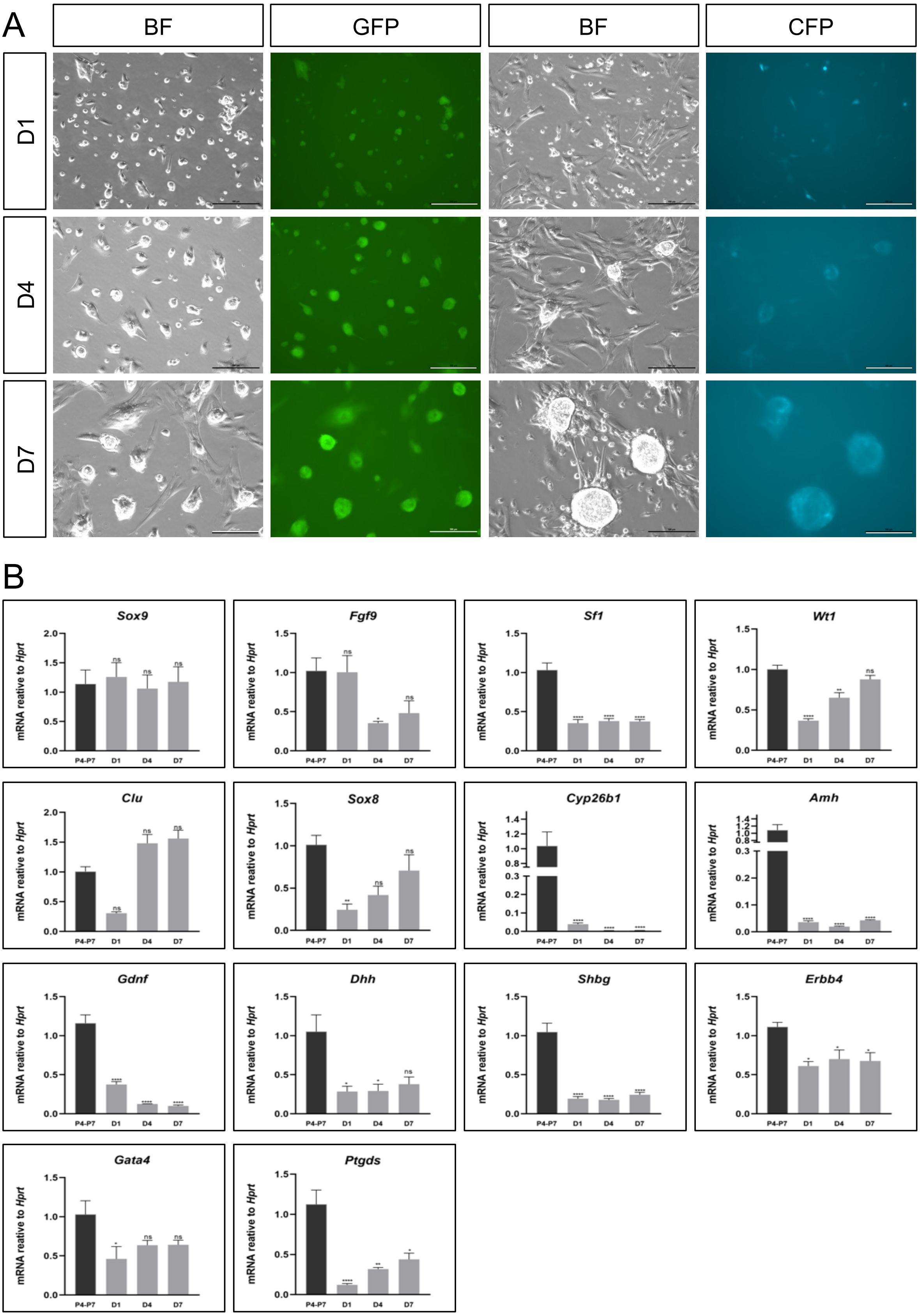

Supplementary Figure 3. Testicular cells cultured in 2D with testis-defined media. (A) BF and fluorescent images of testicular cells harvested from either *Sox9*-IRES-GFP or TESCO-

CFP pups. Cells were cultured for 7 days on 0.2% gelatin-coated dishes in defined media containing Ad-DMEM/F12 media supplemented with testicular growth factors as detailed in Supplementary Table 1. Scale bars, 100 μm. (B) qPCR analysis of testicular cells from TESCO-CFP mice, cultured for 1-7 days with defined media. Data are presented as mean $2^{-\Delta\Delta Ct}$ values ±SEM normalized to the housekeeping gene *Hprt*. **P* < 0.05, ***P* < 0.01, ****P* < 0.001, and *****P* < 0.0001, ns - not significant. *N*=5.

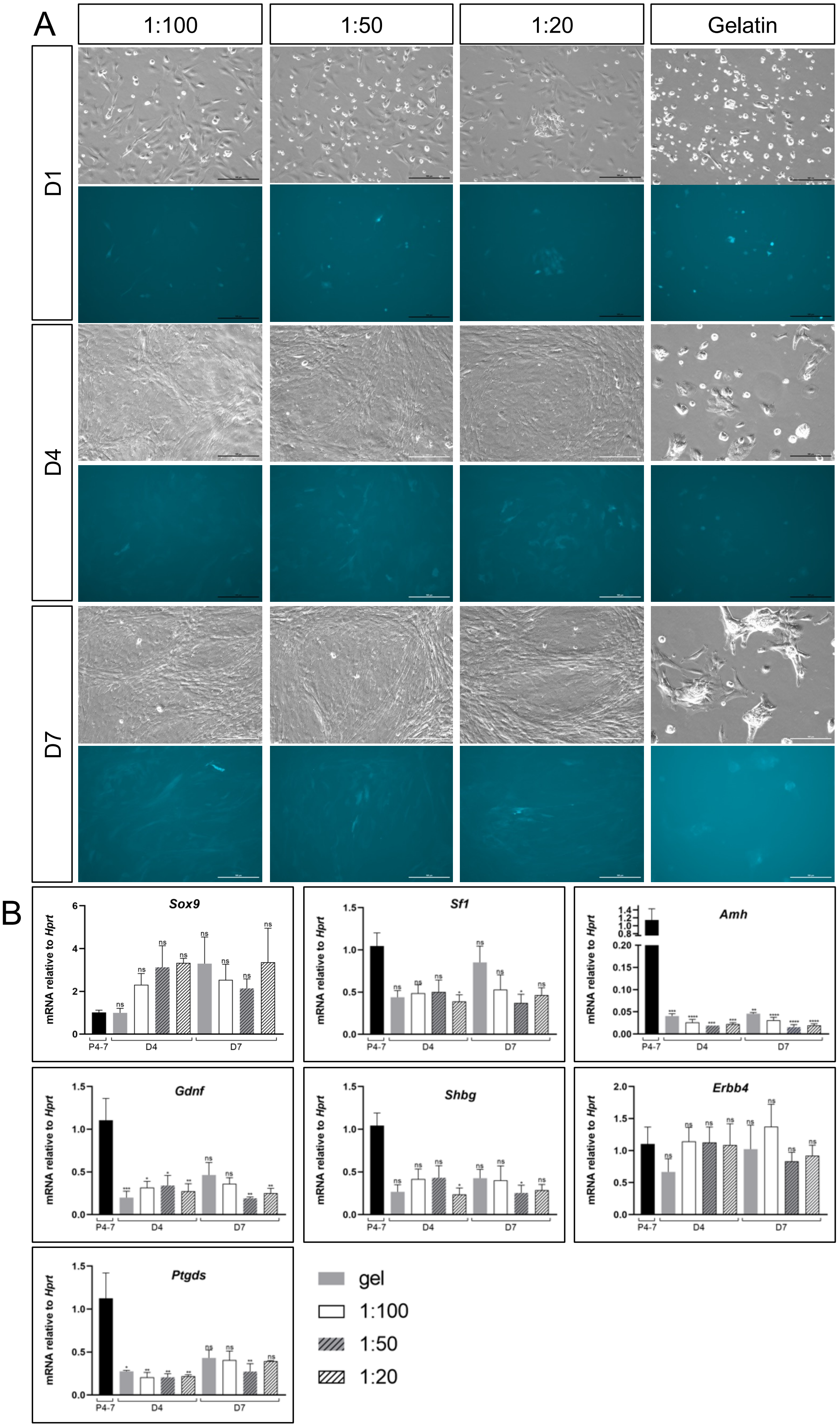
A
1:100
1:50
1:20
Gelatin
D1
D4
D7
B
Sox9
Sf1
Amh
Gdnf
Shbg
Erbb4
Ptgds
mRNA relative to Hprt
P4-7
D4
D7
gel
1:100
1:50
1:20

Supplementary Figure 4. Testicular cells cultured on Geltrex coating in defined media. (A) BF and fluorescent images of testicular cells harvested from TESCO-CFP pups and cultured on various Geltrex dilutions (1:100, 1:50 and 1:20) or 0.2% gelatin (gel) coated wells. Scale bars, 100 µm. (B) qPCR analysis of testicular cells from TESCO-CFP mice, cultured for 1-7 days on defined media and either Geltrex or Gelatin coating. Data are presented as mean $2^{-\Delta\Delta Ct}$ values ±SEM normalized to the housekeeping gene *Hprt*. $^{*}P < 0.05$, $^{**}P < 0.01$, $^{***}P < 0.001$, and $^{****}P < 0.0001$, ns - not significant. N=5.

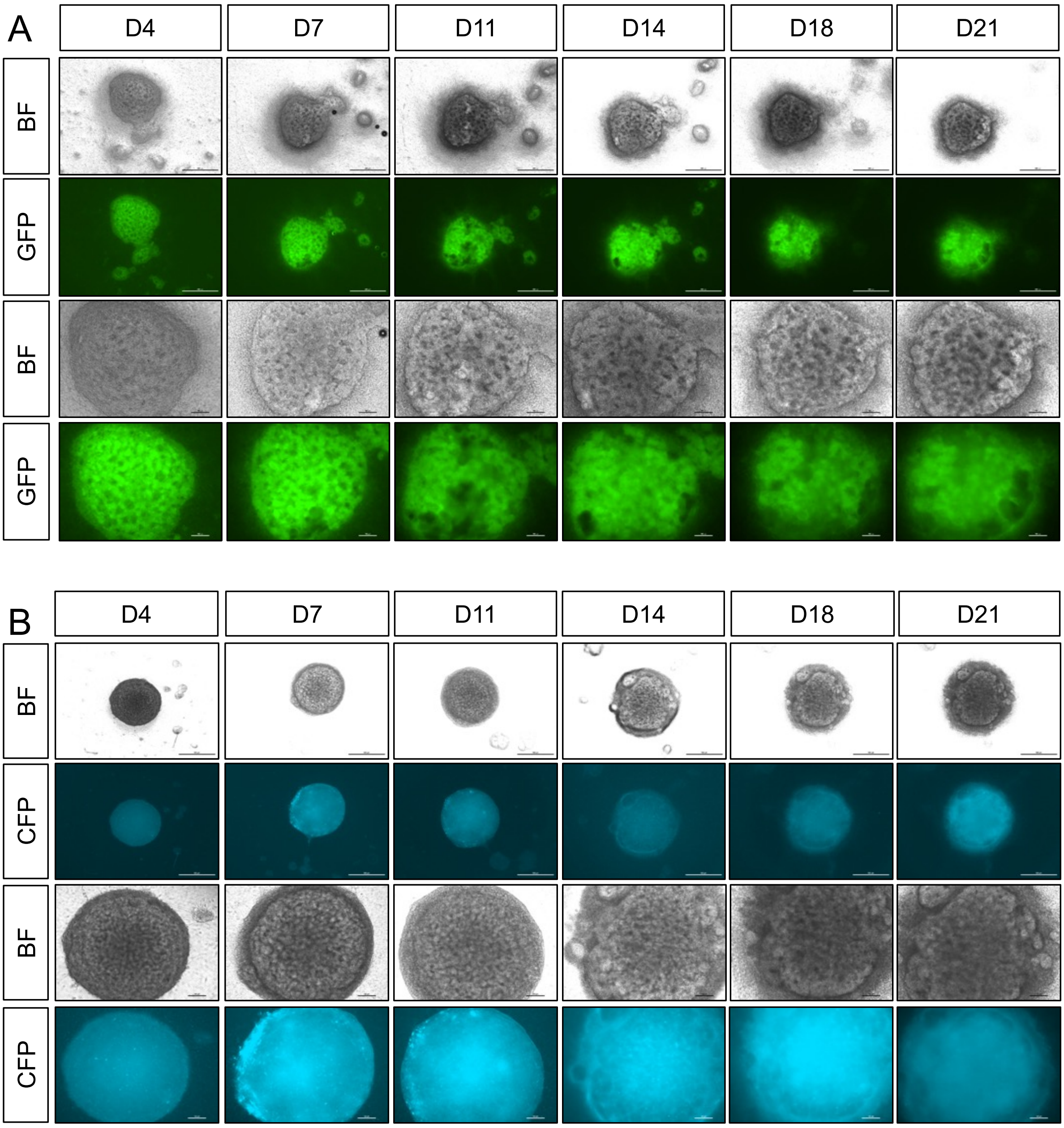

Supplementary Figure 5. Testicular neonatal organoids cultured on transwell inserts. (A) Representative BF and fluorescent images of organoids derived from *Sox9*-IRES-GFP testicular cells and cultured for up to 21 days on transwell inserts. (B) Representative BF and fluorescent images of organoids derived from TESCO-CFP testicular cells and cultured for up to 21 days on transwell inserts. Scale bars, 100 µm.

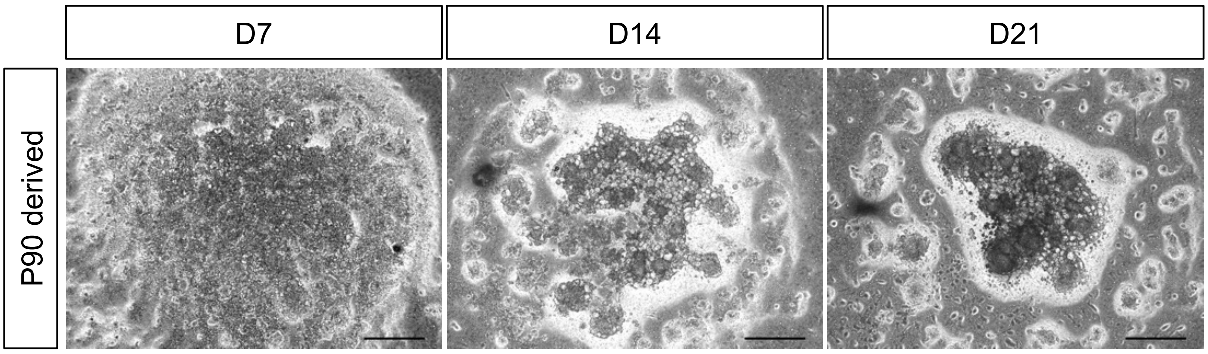

Supplementary Figure 6. Organoids generated from mature testes. Representative BF images of organoids generated from P90 primary testicular cells, cultured for up to 21 days on transwell inserts in defined media. Scale bar, 500 µm.

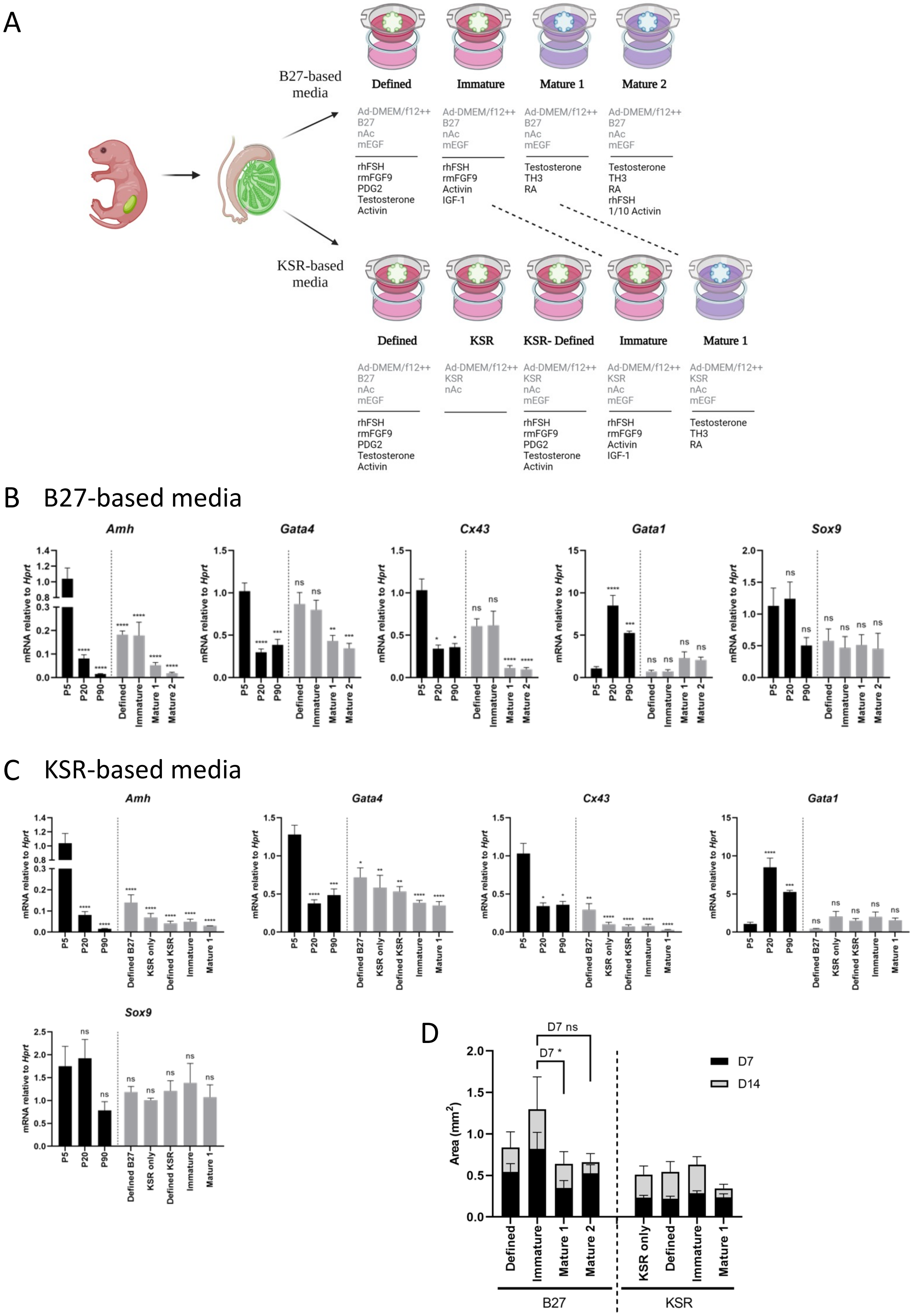
A
B27-based media
Defined
Immature
Mature 1
Mature 2
Ad-DMEM/f12++
B27
nAc
mEGF
rhFSH
rmFGF9
PDG2
Testosterone
Activin
Ad-DMEM/f12++
B27
nAc
mEGF
rhFSH
rmFGF9
Activin
IGF-1
Ad-DMEM/f12++
B27
nAc
mEGF
Testosterone
TH3
RA
Ad-DMEM/f12++
B27
nAc
mEGF
Testosterone
TH3
RA
rhFSH
1/10 Activin
KSR-based media
Defined
KSR
KSR- Defined
Immature
Mature 1
Ad-DMEM/f12++
B27
nAc
mEGF
rhFSH
rmFGF9
PDG2
Testosterone
Activin
Ad-DMEM/f12++
KSR
nAc
Ad-DMEM/f12++
KSR
nAc
mEGF
rhFSH
rmFGF9
PDG2
Testosterone
Activin
Ad-DMEM/f12++
KSR
nAc
mEGF
rhFSH
rmFGF9
Activin
IGF-1
Ad-DMEM/f12++
KSR
nAc
mEGF
Testosterone
TH3
RA
B B27-based media
Amh
Gata4
Cx43
Gata1
Sox9
mRNA relative to Hprt
P5
P20
P90
Defined
Immature
Mature 1
Mature 2
C KSR-based media
Amh
Gata4
Cx43
Gata1
Sox9
mRNA relative to Hprt
P5
P20
P90
Defined B27
KSR only
Defined KSR
Immature
Mature 1
D
D7 ns
D7 *
Area (mm²)
D7
D14
Defined
Immature
Mature 1
Mature 2
KSR only
Defined
Immature
Mature 1
B27
KSR

Supplementary Figure 7. Refinement of media composition for immature and mature state of testicular organoids. (A) Schematic representation of the surveyed media compositions on either B27-based or KSR-based media. The invariable additions are labelled in gray and the growth factors added to each media are labelled in black. For full media composition see Supplementary Table 1. Scheme created using BioRender. (B) qPCR analysis of Sertoli cell markers in organoids cultured in B27-based media for 14 days, compared to P5, P20 and P90 *in vivo* testis. Data are presented as mean $2^{-\Delta\Delta Ct}$ values ±SEM normalized to the housekeeping gene *Hprt*. *$P < 0.05$, **$P < 0.01$, ***$P < 0.001$, and ****$P < 0.0001$, ns - not significant. N=4-5. (C) qPCR analysis of Sertoli cell markers in organoids cultured on KSR-based media for 14 days, compared to P5, P20 and P90 *in vivo* testis. Data are presented as mean $2^{-\Delta\Delta Ct}$ values ±SEM normalized to the housekeeping gene *Hprt*. *$P < 0.05$, **$P < 0.01$, ***$P < 0.001$, and ****$P < 0.0001$, ns - not significant. N=4-5. (D) Organoid area on days 7 and 14 in B27- and KSR-based media. D7 bars are in black, D14 bars are cumulative and shaded grey. Data are presented as mean area ±SEM. Statistical significance is shown for mature 1 and mature 2 vs. immature media at day 7 of culture. *$P < 0.05$, ns- not significant. N=5-8.

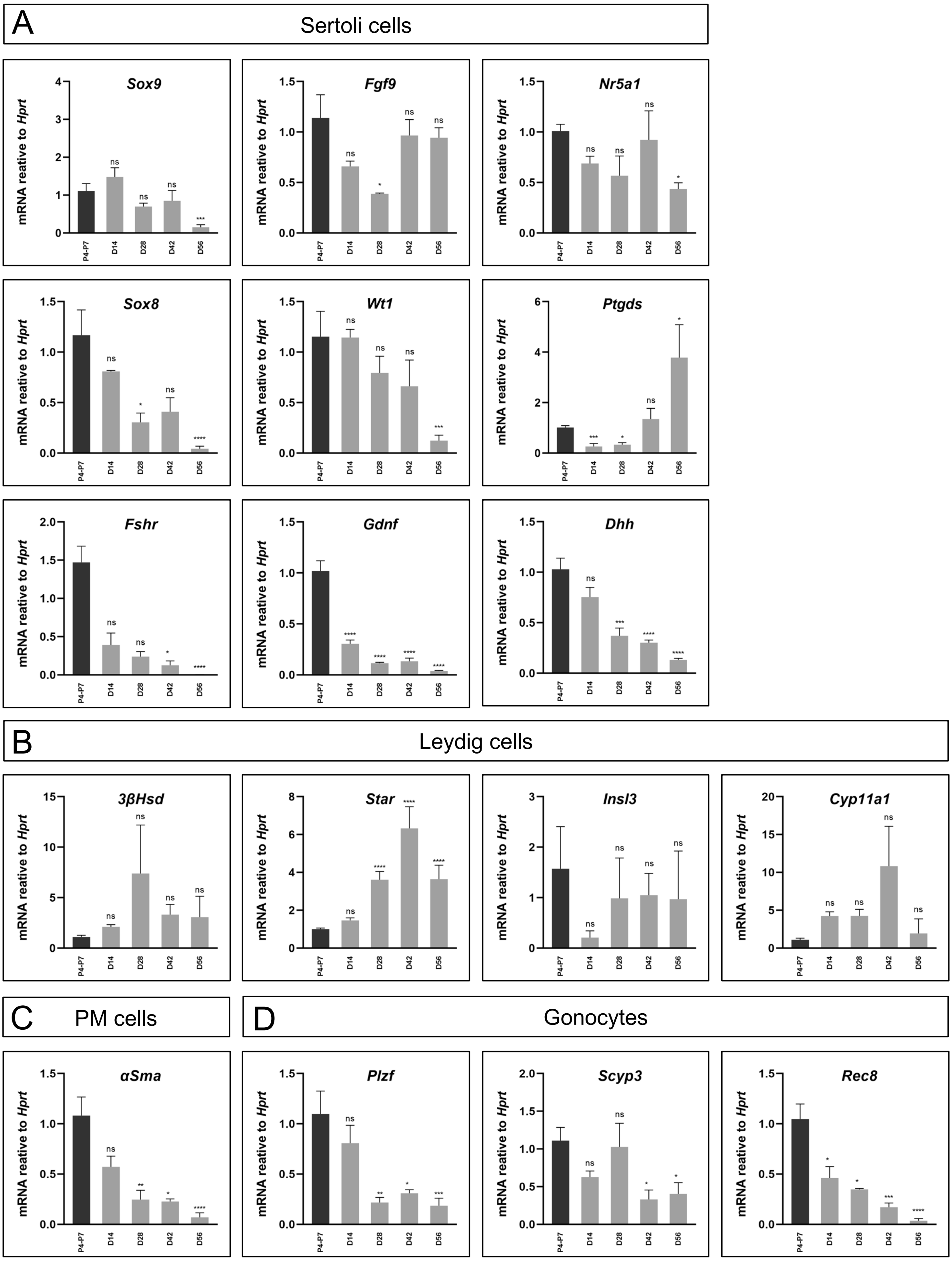

Supplementary Figure 8. Gene expression profiles of organoids cultured in transition media over time. (A-D) Quantitative RT-PCR was performed on mRNA extracted from neonatal testes, or TESCO-CFP organoids cultured for the indicated number of days on transition media assessing Sertoli cell markers (A), Leydig cell markers (B) or other testicular

cells markers (C-D). Gene names are presented in the title. D denotes culture day of organoids, grey. P denotes days postpartum (dpp) of *in vivo* testis, black. Data are presented as mean $2^{-\Delta\Delta Ct}$ values ±SEM normalized to the housekeeping gene *Hprt*. $*P < 0.05$, $**P < 0.01$, $***P < 0.001$, and $****P < 0.0001$, ns- not significant. N=3-6.

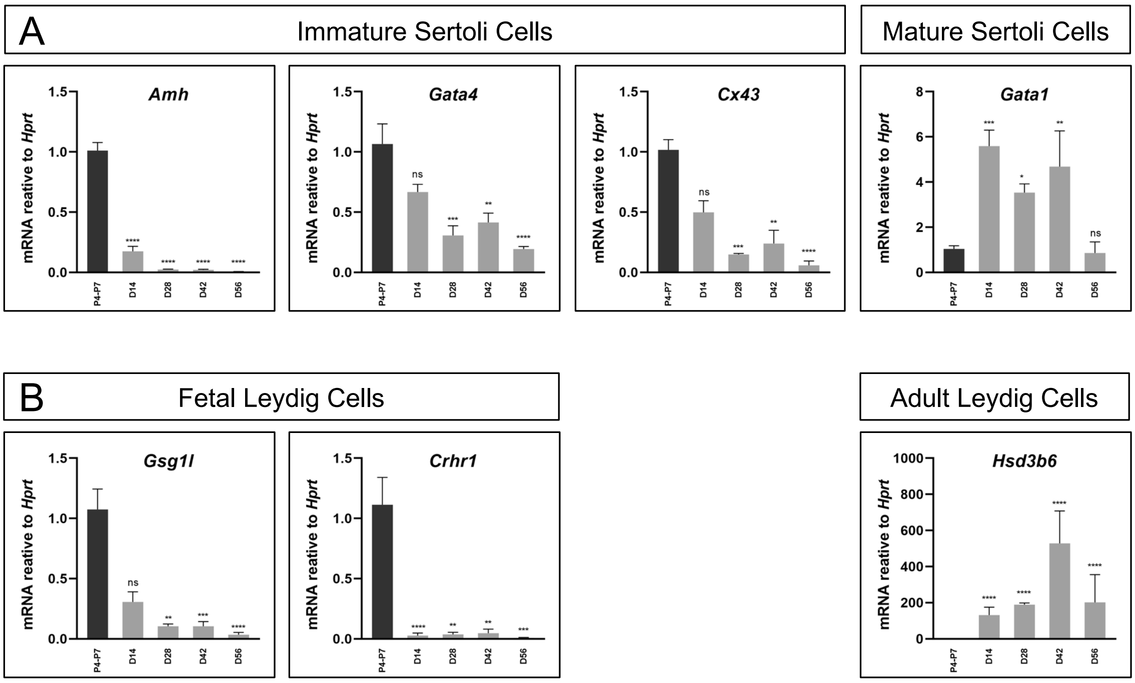

Supplementary Figure 9. Transition media promotes maturation of Sertoli and Leydig cells. (A-B) Quantitative RT-PCR was performed on mRNA extracted from neonatal testes, or TESCO-CFP organoids cultured for the indicated number of days on transition media assessing Sertoli cell immature vs. mature markers (A), or fetal Leydig cell vs. adult Leydig cell markers (B). Gene names are presented in the title. D denotes culture day of organoids, grey. P denotes days postpartum (dpp) of *in vivo* testis, black. Data are presented as mean $2^{-\Delta\Delta Ct}$ values ±SEM normalized to the housekeeping gene *Hprt*. **P* < 0.05, ***P* < 0.01, ****P* < 0.001, and *****P* < 0.0001, ns- not significant. *N*=3-6.

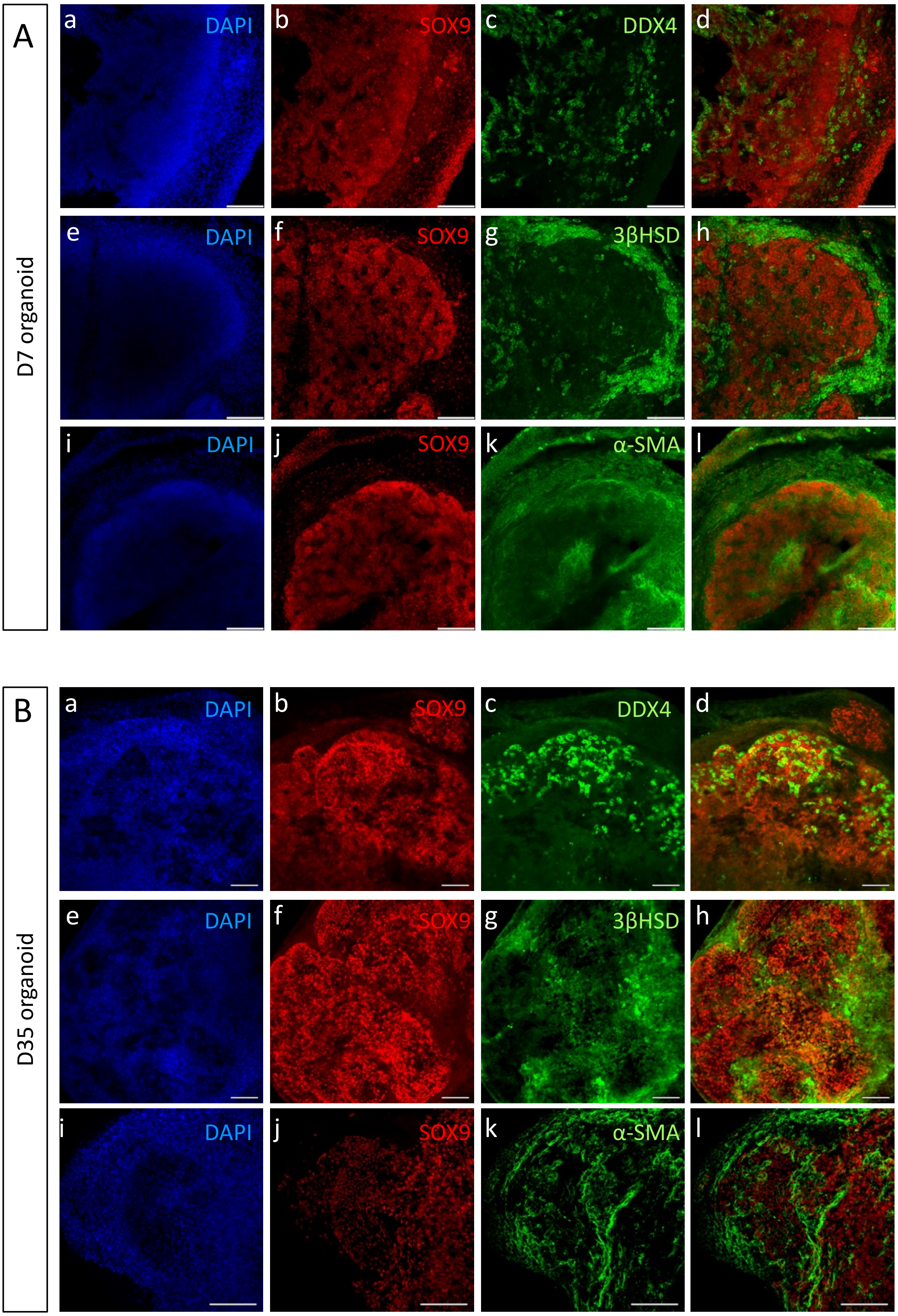
A
D7 organoid
a
DAPI
b
SOX9
c
DDX4
d
e
DAPI
f
SOX9
g
3βHSD
h
i
DAPI
j
SOX9
k
α-SMA
l
B
D35 organoid
a
DAPI
b
SOX9
c
DDX4
d
e
DAPI
f
SOX9
g
3βHSD
h
i
DAPI
j
SOX9
k
α-SMA
l

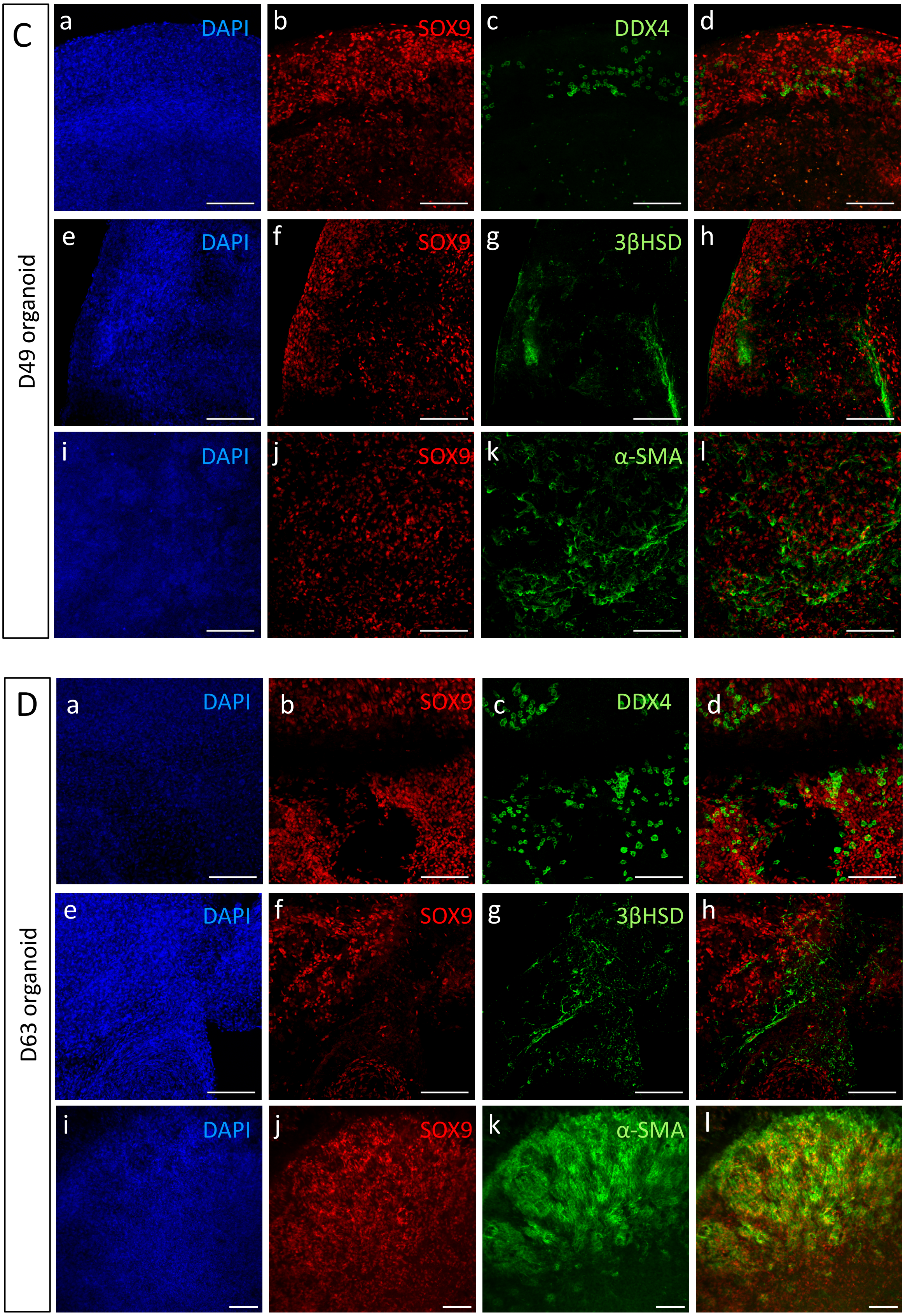
C
D49 organoid
a DAPI
b SOX9
c DDX4
d
e DAPI
f SOX9
g 3βHSD
h
i DAPI
j SOX9
k α-SMA
l
D
D63 organoid
a DAPI
b SOX9
c DDX4
d
e DAPI
f SOX9
g 3βHSD
h
i DAPI
j SOX9
k α-SMA
l

Supplementary Figure 10. Testicular organoids on transition media preserve testicular structures and gonadal cell types for up to 9 weeks. (A-D) Whole mount co-immunostaining of markers for the major types of testicular cells in organoids cultured for 7 days (A), 35 days (B), 49 days (C) and 63 days (D). SOX9 marks Sertoli cells, DDX4 labels gonocytes, 3ßHSD marks Leydig cells and α-SMA labels PM cells. Images on the right are merged views of all channels. Scale bars, 100 µm.

	Table S1. Media composition				
	**Media**	**Component**	**Final concentration**	**Manufacturer**	**Cat. number**
	**Serum based medium**	Ad-DMEM-F12	/	Thermo Fisher Scientific	12634010
		Penicillin-Streptomycin	1x	Thermo Fisher Scientific	15140122
		L-glutamine	2mM	Biological industries	03-020
		Fetal Bovine Serum (FBS)	2%	Thermo Fisher Scientific	10270106
**B27-based media**	**Defined medium**	Ad-DMEM-F12	/	Thermo Fisher Scientific	12634010
		Penicillin-Streptomycin	1x	Thermo Fisher Scientific	15140122
		L-glutamine	2mM	Biological industries	03-020
		B-27 supplement	1X	Thermo Fisher Scientific	17504044
		N-Acetyl-L-cysteine	1.25mM	Sigma	A9165
		Mouse EGF	50ng/ml	Thermo Fisher Scientific	PMG8043
		Recombinant human FSH	22ng/ml	R&D Systems	5925-FS
		Recombinant mouse FGF9	50ng/ml	R&D Systems	7399-F9
		Prostaglandin D2 (PDG2)	0.5µg/ml	Cayman	12010
		Testosterone	1µM	Sigma	T1500
		Recombinant Human/Mouse/Rat Activin A	50ng/ml	R&D Systems	338- AC
	**Immature**	Ad-DMEM-F12	/	Thermo Fisher Scientific	12634010
		Penicillin-Streptomycin	1x	Thermo Fisher Scientific	15140122
		L-glutamine	2mM	Biological industries	03-020-1A
		B-27 supplement	1X	Thermo Fisher Scientific	17504044
		N-Acetyl-L-cysteine	1.25mM	Sigma	A9165
		Mouse EGF	50ng/ml	Thermo Fisher Scientific	PMG8043
		Recombinant human FSH	22ng/ml	R&D Systems	5925-FS
		Recombinant mouse FGF9	50µg/ml	R&D Systems	7399-F9
		Recombinant Human/Mouse/Rat Activin A	50ng/ml	R&D Systems	338- AC
		Recombinant human IGF-1	100ng/ml	R&D Systems	291-G1
	**Mature1**	Ad-DMEM-F12	/	Thermo Fisher Scientific	12634010
		Penicillin-Streptomycin	1x	Thermo Fisher Scientific	15140122
		L-glutamine	2mM	Biological industries	03-020-1A
		B-27 supplement	1X	Thermo Fisher Scientific	17504044
		N-Acetyl-L-cysteine	1.25mM	Sigma	A9165
		Mouse EGF	50ng/ml	Thermo Fisher Scientific	PMG8043
		Testosterone	1µM	Sigma	T1500
		Thyroid hormone (T3)	100nM	Tocris	6666
		Retinoic acid	1µM	Sigma	R2625
	**Mature2**	Ad-DMEM-F12	/	Thermo Fisher Scientific	12634010
		Penicillin-Streptomycin	1x	Thermo Fisher Scientific	15140122
		L-glutamine	2mM	Biological industries	03-020-1A
		B-27 supplement	1X	Thermo Fisher Scientific	17504044
		N-Acetyl-L-cysteine	1.25mM	Sigma	A9165
		Mouse EGF	50ng/ml	Thermo Fisher Scientific	PMG8043
		Recombinant human FSH	22ng/ml	R&D Systems	5925-FS
		Testosterone	1µM	Sigma	T1500
		Thyroid hormone (T3)	100nM	Tocris	6666
		Retinoic acid	1µM	Sigma	R2625
		Recombinant Human/Mouse/Rat Activin A	5ng/ml	R&D Systems	338- AC
**KSR-based media**	**KSR**	Ad-DMEM-F12	/	Thermo Fisher Scientific	12634010
		Penicillin-Streptomycin	1x	Thermo Fisher Scientific	15140122
		L-glutamine	2mM	Biological industries	03-020-1A
		KnockOut Serum Replacement (KSR)	10%	Thermo Fisher Scientific	10828028
		N-Acetyl-L-cysteine	1.25mM	Sigma	A9165
	**KSR - Defined**	Ad-DMEM-F12	/	Thermo Fisher Scientific	12634010
		Penicillin-Streptomycin	1x	Thermo Fisher Scientific	15140122
		L-glutamine	2mM	Biological industries	03-020-1A
		KnockOut Serum Replacement (KSR)	10%	Thermo Fisher Scientific	10828028
		N-Acetyl-L-cysteine	1.25mM	Sigma	A9165
		Mouse EGF	50ng/ml	Thermo Fisher Scientific	PMG8043
		Recombinant human FSH	22ng/ml	R&D Systems	5925-FS
		Recombinant mouse FGF9	50µg/ml	R&D Systems	7399-F9
		Prostaglandin D2 (PDG2)	0.5µg/ml	Cayman	12010
		Testosterone	1µM	Sigma	T1500
		Recombinant Human/Mouse/Rat Activin A	50ng/ml	R&D Systems	338- AC
	**Immature**	Ad-DMEM-F12	/	Thermo Fisher Scientific	12634010
		Penicillin-Streptomycin	1x	Thermo Fisher Scientific	15140122
		L-glutamine	2mM	Biological industries	03-020-1A
		KnockOut Serum Replacement (KSR)	10%	Thermo Fisher Scientific	10828028
		N-Acetyl-L-cysteine	1.25mM	Sigma	A9165
		Mouse EGF	50ng/ml	Thermo Fisher Scientific	PMG8043
		Recombinant human FSH	22ng/ml	R&D Systems	5925-FS
		Recombinant mouse FGF9	50µg/ml	R&D Systems	7399-F9
		Recombinant Human/Mouse/Rat Activin A	50ng/ml	R&D Systems	338- AC
		Recombinant human IGF-1	100ng/ml	R&D Systems	291-G1
	**Mature1**	Ad-DMEM-F12	/	Thermo Fisher Scientific	12634010
		Penicillin-Streptomycin	1x	Thermo Fisher Scientific	15140122
		L-glutamine	2mM	Biological industries	03-020-1A
		KnockOut Serum Replacement (KSR)	10%	Thermo Fisher Scientific	10828028
		N-Acetyl-L-cysteine	1.25mM	Sigma	A9165
		Mouse EGF	50ng/ml	Thermo Fisher Scientific	PMG8043
		Testosterone	Sigma	T1500	T1500
		Thyroid hormone (T3)	Tocris	6666	6666
		Retinoic acid	Sigma	R2625	R2625

Table S2. Primers for genotyping mice			
**Primer name**	**Description**	**Sequence 5' to 3'**	**Product size**
CFP-F	CFP	CGTGACCACCCTGACCTGG	327 bp product
CFP-R	CFP	GTGGCGGATCTTGAAGTTGG	
GFP-F	GFP	TGCAGTGCTTCAGCCGCTAC	462 bp product
GFP-R	GFP	CCAGCAGGACCATGTGATCG	

Table S3. Primers for Real-time quantitative RT-PCR			
**Primer name**	**Gene**	**Marker of**	**Sequence 5' to 3'**
m3βHsd_F	*3βHsd*	*Leydig cells*	CTCAGTTCTTAGGCTTCAGCAATTAC
m3βHsd_R			CCAAAGGCAAGATATGATTTAGGA
mAcrosin_F	*Acrosin*	*Mature sperm*	TGTCCGTGGTTGCCAAGGATAACA
mAcrosin_R			AATCCGGGTACCTGCTTGTGAGTT
mAmh_F	*Amh*	*Immature Sertoli cells*	CCACGGTTAGCACCAAATAGC
mAmh_R			CACACAGAACCTCTGCCCTACTC
mAr_F	*Ar*	*Sertoli cells*	CTGGGAAGGGTCTACCCAC
mAr_R			GGTGCTATGTTAGCGGCCTC
mClu_F	*Clu*	*Sertoli cells*	TGAAGGGCCAGTGTGAAAAGT
mClu_R			TTGAACAGTCCACAGACAAGATCTC
mCrhr1_F	*Crhr1*	*Fetal Leydig cells*	CCAGGATCAGCAGTGTGAGA
mCrhr1_R			TGTTTGTGGTGTTGTAGCGG
mCx43_F	*Cx43*	*Adult Sertoli cell*	GGTCTGAGAGCCCGAACTCTCCT
mCx43_R			CCCATGTCTGGGCACCTCTCTTT
mCyp11a1_F	*Cyp11a1*	*Leydig cells*	CACTTCTGGAGGGAGAGTGG
mCyp11a1_R			ATGCCTGGAAGAAAGACCGA
mCyp26b1_F	*Cyp26b1*	*Sertoli cells/ FLC*	AAGGGCTCCATGGGATTC
mCyp26b1_R			ACGACTGGAAGCCGGAAC
mDhh_F	*Dhh*	*Sertoli cells*	CCGCAACCACATCCACGTA
mDhh_R			CGGACCGCCAGTGAGTTATC
mErbb4_F	*Erbb4*	*Sertoli cells*	CAGCGCTTCTCAGTCAGTGT
mErbb4_R			CTGCTGTTCCAGGTCAGAGA
mFgf9_F	*Fgf9*	*Sertoli cells*	TCTTCCCCAACGGTACTATCCA
mFgf9_R			CGAAGCGGCTGTGGTCTTT
mFshr_F	*Fshr*	*Sertoli cells*	TTGCCTGATGATGTTTTCCA
mFshr_R			CTGGCCCTCAACTTCTTCAG
mGata1_F	*Gata1*	*Adult Sertoli cell*	TGGGGACCTCAGAACCCTTG
mGata1_R			GGCTGCATTTGGGGAAGTG
mGata4_F	*Gata4*	*Sertoli cells*	CCCCAATCTCGATATGTTTGATG
mGata4_R			TTGACACACTCTCTGCCTTCTGA
mGdnf_F	*Gdnf*	*Sertoli cells*	GGGTGCGTTTTAACTGCCATA
mGdnf_R			GCCCAAACCCAAGTCAGTGA
mGsg1l_F	*Gsg1l*	*Fetal Leydig cells*	GAGGAGCCGACGTTCATAGA
mGsg1l_R			AGCACTGACGGTTTAGCTCT
mHprt_F	*Hprt*	*HK gene*	GCAGTACAGCCCCAAAATGG
mHprt_R			GGTCCTTTTCACCAGCAAGCT
mHsd3b6_F	*Hsd3b6*	*Adult Leydig cells*	GGAGGAGATCAGGGTCCTGG
mHsd3b6_R			CTAGGATGGTCTGCCTGGG
mInsl3_F	*Insl3*	*Leydig cells*	GCTGCTACTGATGCTCCTGG
mInsl3_R			CTAGACCCGCAGCTTCCTC
mNr5a1_F	*Nr5a1*	*Sertoli cells*	CCTCGATGTGAAATTCCTGAACA
mNr5a1_R			TCCTGGGCGTCCTTTACG
mPlzf_F	*Plzf*	*Spermatogonial stem cells (SSC)*	CCAGTTGGAGACGACCTACA
mPlzf_R			GTGGCAGAGTTTGCACTCAA
mPtgds_F	*Ptgds*	*Sertoli cells*	GGCTCCTGGACACTACACCT
mPtgds_R			CATAGTTGGCCTCCACCACT
mRec8_F	*Rec8*	*Meiotic spermatids*	GCCCTAGAAGGTGCTGGTTTGG
mRec8_R			GTGGGGTCACCTCAGTGAGTAGG
mScyp3_F	*Scyp3*	*Meiotic spermatids*	GCAGTCTAGAATTGTTCAGAGCCAGA
mScyp3_R			TCCAAACTCTTTATGAACTGCTCGTG
mShbg_F	*Shbg*	*Sertoli cells*	CCAAAATCAGCAAACCCCATT
mShbg_R			CTGGATCCCAGGTTCGAAAC
mSox8_F	*Sox8*	*Sertoli cells*	AGCGAGAAGAGGCCGTTTG
mSox8_R			TCAGTACCAGAGTCTGAGTCG
mSox9_F	*Sox9*	*Sertoli cells*	AAGAAAGACCACCCCGATTACA
mSox9_R			CAGCGCCTTGAAGATAGCATT
mStar_F	*Star*	*Leydig cells*	GACGTCGGAGCTCTCTGCTT
mStar_R			GCCTTCTGCATAGCCACCTC
mWt1_F	*Wt1*	*Sertoli cells*	TTGAATGCATGACCTGGAATCA
mWt1_R			TTCCCTTTAAGGTAGCTCCTAGGTT
mαSma_F	*αSma*	*Peritubular myoid cells (PMC)*	CCCCTGAAGAGCATCCGACA
mαSma_R			TGGCGGGGACATTGAAGGT

Table S4. Antibody list				
**Protein**	**Cat. No.**	**Raised in**	**Company**	**Dilution**
SOX9	AB5535	Rabbit	Merck Millipore	1:5000
DDX4	ab27591	Mouse	Abcam	1:300
αSMA	A2547	Mouse	Sigma	1:200
3βHSD	sc-515120	Mouse	Santa Cruz	1:200
γH2AX	ab11174	Rabbit	Abcam	1:1000
TRA98	ab82527	Rat	Abcam	1:200
AMH	sc-6886	Goat	Santa Cruz	1:200
CLAUDIN-11	Zy-364500	Rabbit	Invitrogen	1:100
REC8	ab192241	Rabbit	Abcam	1:200

Movie S1. Supplementary movie 1_D21 organoid_SOX9_DDX4 b_d

Movie S2. Supplementary movie 2_D21 organoid_SOX9_3bHSD f_h

Movie S3. Supplementary movie 3_D21 organoid_SOX9_SMA j_l

Movie S4. Supplementary movie 4_D21 organoid_AMH_CLD11_DDX4 m_p

Movie S5. Supplementary movie 5_D21 organoid_SOX9

Movie S6. Supplementary movie 6_D21 organoid_DAPI
